# Mutagenesis Mapping of RNA Structures within the Foot-and-Mouth Disease Virus Genome Reveals Functional Elements Localized in the Polymerase (3D^pol^)-Encoding Region

**DOI:** 10.1128/mSphere.00015-21

**Published:** 2021-07-14

**Authors:** Lidia Lasecka-Dykes, Fiona Tulloch, Peter Simmonds, Garry A. Luke, Paolo Ribeca, Sarah Gold, Nick J. Knowles, Caroline F. Wright, Jemma Wadsworth, Mehreen Azhar, Donald P. King, Tobias J. Tuthill, Terry Jackson, Martin D. Ryan

**Affiliations:** a The Pirbright Institute, Pirbright, Surrey, United Kingdom; b Biomedical Sciences Research Complex (BSRC), School of Biology, University of St. Andrews, St. Andrews, United Kingdom; c Nuffield Department of Experimental Medicine, University of Oxford, Oxford, United Kingdom; University of Zurich

**Keywords:** CDLR-based mutagenesis, RNA structure, bioinformatics, foot-and-mouth disease virus (FMDV), viral replication

## Abstract

RNA structures can form functional elements that play crucial roles in the replication of positive-sense RNA viruses. While RNA structures in the untranslated regions (UTRs) of several picornaviruses have been functionally characterized, the roles of putative RNA structures predicted for protein coding sequences (or open reading frames [ORFs]) remain largely undefined. Here, we have undertaken a bioinformatic analysis of the foot-and-mouth disease virus (FMDV) genome to predict 53 conserved RNA structures within the ORF. Forty-six of these structures were located in the regions encoding the nonstructural proteins (nsps). To investigate whether structures located in the regions encoding the nsps are required for FMDV replication, we used a mutagenesis method, CDLR mapping, where sequential coding segments were shuffled to minimize RNA secondary structures while preserving protein coding, native dinucleotide frequencies, and codon usage. To examine the impact of these changes on replicative fitness, mutated sequences were inserted into an FMDV subgenomic replicon. We found that three of the RNA structures, all at the 3′ termini of the FMDV ORF, were critical for replicon replication. In contrast, disruption of the other 43 conserved RNA structures that lie within the regions encoding the nsps had no effect on replicon replication, suggesting that these structures are not required for initiating translation or replication of viral RNA. Conserved RNA structures that are not essential for virus replication could provide ideal targets for the rational attenuation of a wide range of FMDV strains.

**IMPORTANCE** Some RNA structures formed by the genomes of RNA viruses are critical for viral replication. Our study shows that of 46 conserved RNA structures located within the regions of the foot-and-mouth disease virus (FMDV) genome that encode the nonstructural proteins, only three are essential for replication of an FMDV subgenomic replicon. Replicon replication is dependent on RNA translation and synthesis; thus, our results suggest that the three RNA structures are critical for either initiation of viral RNA translation and/or viral RNA synthesis. Although further studies are required to identify whether the remaining 43 RNA structures have other roles in virus replication, they may provide targets for the rational large-scale attenuation of a wide range of FMDV strains. FMDV causes a highly contagious disease, posing a constant threat to global livestock industries. Such weakened FMDV strains could be investigated as live-attenuated vaccines or could enhance biosecurity of conventional inactivated vaccine production.

## INTRODUCTION

The genomes of RNA viruses not only encode proteins but also contain nontemplated functional elements in both the coding and untranslated regions (UTRs). These elements can be secondary or higher-order RNA structures such as simple stem-loops or more complex structures which include pseudoknots and so-called kissing loops that mediate long-range RNA-RNA interactions (1–12). The function, shape, and number of such RNA functional elements are often characteristic for a particular group of viruses, where they play important roles in processes such as the initiation of viral RNA translation and replication, subgenomic mRNA transcription, frameshift events, viral RNA encapsidation, and modulation of host’s antiviral responses (reviewed in reference 13). Since many RNA viruses are of medical and veterinary importance, characterization of these RNA structures brings us closer to understanding viral pathogenicity and provides opportunities for disease control.

Foot-and-mouth disease virus (FMDV) is the causative agent of foot-and-mouth disease (FMD), a highly contagious disease of cloven-hoofed animals (including livestock) (reviewed in reference 14). FMD is endemic in Africa and Asia, where it impacts upon productivity and trade as well as posing a constant threat of causing costly incursions into disease-free countries (15–21). Control of FMD by vaccination in endemic settings is complicated by the high antigenic variability of the seven serotypes of FMDV: A, Asia 1, C (not reported since 2004), O, Southern African Territories 1 (SAT 1), SAT 2, and SAT 3 (19, 20, 22–24).

FMDV is a small nonenveloped positive-sense single-stranded RNA virus classified in the species *Foot-and-mouth disease virus*, genus *Aphthovirus* in the family *Picornaviridae*. The FMDV genome is ∼8.5 kb in size and composed of a single, long open reading frame (ORF) which is flanked by 5′ and 3′ UTRs (reviewed in reference 25). The encoded polyprotein is co- and posttranslationally cleaved by viral proteases (L^pro^ and 3C^pro^) and by a ribosomal skipping event mediated by the 2A peptide into a number of functional precursors and the mature proteins (26–34). The coding sequence for the FMDV ORF is arbitrarily divided into four regions (5′-L^pro^, P1, P2, and P3-3′). The P1 region encodes the capsid proteins (1A, 1B, 1C, and 1D, also called VP4, VP2, VP3, and VP1, respectively), while the P2 and P3 regions encode the nonstructural proteins (nsps) (reviewed in reference 25).

There are several RNA structures within picornavirus genomes that have been bioinformatically predicted and characterized biochemically (12, 35–39). These structures are predominantly located in the UTRs and have been shown to be important for replication and translation of picornavirus genomes (reviewed in reference 40). Within the 5′ UTR of the FMDV genome, the S-fragment forms a single, long hairpin structure (293 to 381 nucleotides [nt] in length) and has been reported to play a role in viral replication and innate immune modulation (41–45). Elsewhere in the 5′ UTR, the presence of multiple (two to four) pseudoknots downstream of the poly(C) tract has been shown to determine virus tropism (41, 46). Other key and well-characterized RNA structural elements include a type II internal ribosome entry site (IRES), which initiates cap-independent translation of the viral genome (41, 47–50), while the *cis*-acting replication element (*cre*) acts as a template for uridylylation of the VPg (3B) protein, which then acts as a primer for synthesis of viral RNA (51, 52). The 3′ UTR of the FMDV genome is located upstream of the poly(A) tract and contains two RNA stem-loop structures called SL1 and SL2. These stem-loops interact nonsimultaneously with the S-fragment and IRES, forming long-range interactions that have been shown to be necessary for viral RNA replication (43, 53, 54).

A number of other secondary RNA structures have been predicted computationally to be present within the FMDV ORF (12). However, with the exception of packaging signals (55), the role(s) of these structures in the FMDV replication cycle has not been determined. In this study, we have identified 46 evolutionarily conserved RNA structures within the regions of the FMDV ORF that encode the nsps. Mutagenesis of these structures identified three novel RNA stem-loops in the coding region of the RNA-dependent RNA polymerase (3D^pol^) that are essential for replication of an FMDV subgenomic replicon, suggesting that these structures are required for either initiation of viral RNA translation and/or viral RNA synthesis. In contrast, mutagenesis of the remaining 43 structures had no effect on replicon replication. This approach can aid in the identification of critical viral RNA structures required for viral genome replication and also help identify conserved RNA structures that are not essential for virus replication that could provide ideal targets for the rational attenuation of a wide range of FMDV strains.

## RESULTS

### Prediction of conserved RNA structures within the FMDV genome.

While previous studies have provided evidence that the FMDV genome is highly structured with conserved RNA base pairing throughout the coding part of the genome (12, 56), these studies were conducted on a relatively small data set. Since the number of full genome sequences available on public databases has greatly increased in recent years, before conducting functional studies, we revisited these analyses to predict conserved RNA stem-loops that were common in 118 representative genomic sequences covering all FMDV serotypes (see Table S1 in the supplemental material).

10.1128/mSphere.00015-21.8 TABLE S1 FMDV sequences selected from GenBank. Download Table S1, PDF file, 0.02 MB. © Crown copyright 2021. 2021 Crown https://creativecommons.org/licenses/by/4.0/ This content is distributed under the terms of the Creative Commons Attribution 4.0 International license.

The degree of RNA structure formation in the FMDV genome was estimated bioinformatically through calculation of minimum free energies (MFEs) of the most thermodynamically stable *in vitro* folded RNA sequences (57–59). However, MFE values on their own do not suffice to demonstrate the existence of biologically relevant structures, because they are conditioned by compositional factors, such as G+C content and dinucleotide representation in addition to base order. To account for this, the mean folding energy difference (MFED) metric was developed (60–62), in which MFE values of native sequences are compared to those randomized in base order but preserving mono- and dinucleotide frequencies (the algorithm NDR in the current study [56]). High MFED values are indicative of regions with base order-dependent and potentially biologically relevant RNA secondary structure.

**FIG 1:**
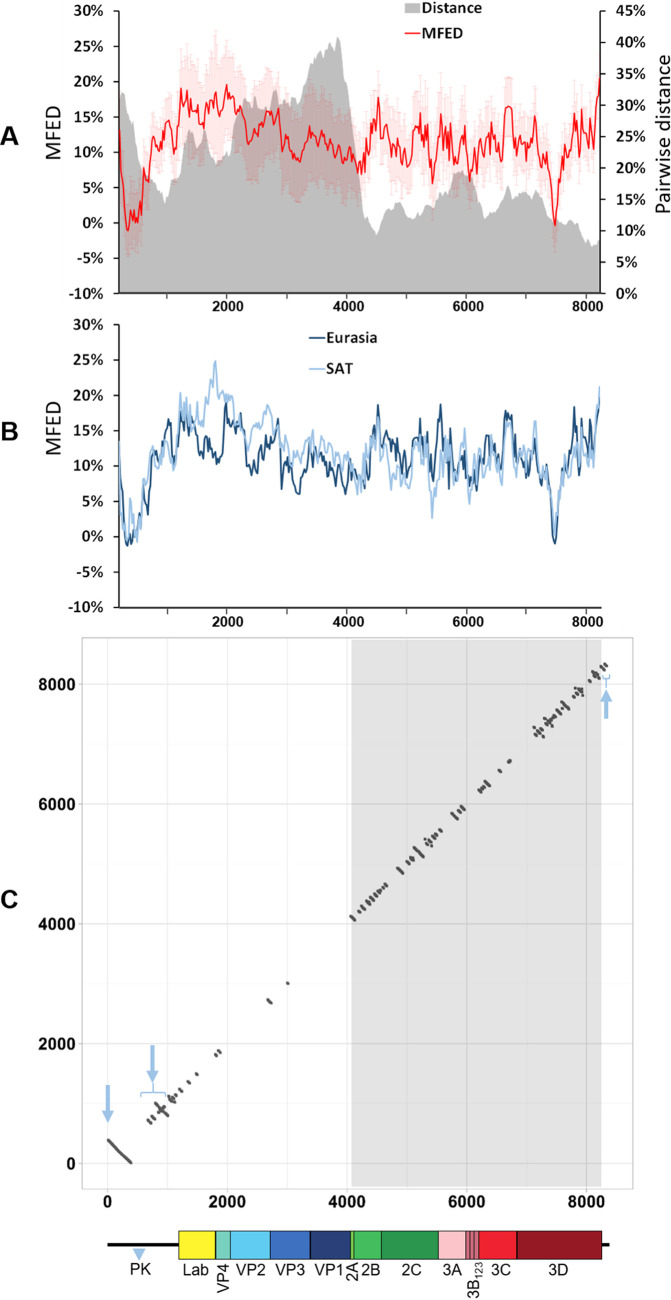
Extent of the conserved RNA secondary structures within the FMDV genome. (A) A scan of pairwise distance (in gray) and mean folding energy difference (MFED) (in red) prepared using SSE v1.4 software and 118 FMDV sequences representing all seven serotypes. The mean values for successive 400-nt fragments across the genome are plotted (where each 400-nt segment overlapped its neighbors by 380 nt). The light red shading represents error bars showing standard deviations from the mean for each MFED data point. The *y* axis shows genome nucleotide position. (B) MFED values for the same FMDV genomic sequences but grouped into Eurasian (A, Asia 1, C, and O serotypes) (dark blue) or SAT (SAT 1 to 3 serotypes) (light blue) clusters. (C) A dot plot graphical representation of RNA structures that were conserved across all seven FMDV serotypes. The *x* axis and *y* axis represent FMDV genome positions, with each dot representing a single pairing between two nucleotides, one with its position marked on the *x* axis and the other one with its position marked on the *y* axis. The three pale blue arrows indicate the locations of the S-fragment, *cre* plus IRES, and SL1 plus SL2 structures on the dot plot graph, respectively (for a detailed visualization of these structures, see Fig. S1 in the supplemental material). The blue triangle marked PK indicates the genomic region containing pseudoknot structures which was excluded from these analyses. The area corresponding to the regions encoding the nonstructural proteins (i.e., P2 and P3) is highlighted in gray. For clarity, a schematic representation of the FMDV genome is drawn to scale.

Using this approach, all 118 FMDV genomic sequences in their genomic (positive-sense) orientations were scanned using the program Fold Energy Scan in the SSE package (63) to calculate MFED values. This analysis employed an incremental sliding window computation with user-defined window size and increment (63) (in our case, 400 and 20 nt, respectively, where each 400-nt segment overlapped its neighbors by 380 nt). Despite the high genomic sequence diversity across all seven serotypes (20% mean nucleotide pairwise distance [± 9% standard deviation {SD}], with 31% [± 5% SD] and 14% [± 7% SD]) average pairwise distance in the regions encoding the capsid proteins and the nsps, respectively), all the FMDV genomes analyzed showed high folding energies across most of their sequence compared to the NDR-permuted controls (Fig. 1A). This indicates that all FMDV sequences possess similar extents of sequence order-dependent RNA secondary structure. To confirm this, full genome sequences were grouped into those of Eurasian (A, Asia 1, C, and O serotypes) and SAT (SAT 1 to 3 serotypes) origin, and average MFED values were determined along the genome for each group. Although we recognize that the grouping may not completely accommodate the interserotypic history of these viruses (genomes of several isolates of SAT serotypes contain 5′ UTR and regions encoding nsps which are phylogenetically more closely related to corresponding genomic regions of Eurasian isolates than of other SAT isolates) (45), the MFED plots showed similar patterns of high and low MFED values across the genome. MFED values were better correlated between FMDV groups in the UTRs and the regions encoding the nsps, identifying a potentially greater degree of RNA structure conservation compared to the more genetically divergent region encoding the capsid proteins (Fig. 1B).

10.1128/mSphere.00015-21.1 FIG S1 Conserved whole-genome RNA structure was predicted for 118 FMDV field isolates as described in the legend to Fig. 1 and regions of previously published structures located within the 5′ UTR (S-fragment, *cre*, and IRES) or 3′ UTR (SL1 and SL2) were visualized using the Forna web service. Download FIG S1, PDF file, 0.2 MB. © Crown copyright 2021. 2021 Crown https://creativecommons.org/licenses/by/4.0/ This content is distributed under the terms of the Creative Commons Attribution 4.0 International license.

The window size used for MFED scanning does not identify individual RNA structures and only highlights regions with high folding energies (which may contain dissimilar structures and/or structures located at different positions). Therefore, RNAalifold program, implemented in the ViennaRNA Package (64), was used to identify individual conserved RNA stem-loops for all 118 whole genomic sequences and, separately, also for the sequences grouped into each FMDV serotype individually. Stem-loops conserved for all seven serotypes were visualized as a dot plot graph, plotting each nucleotide pairing (represented by individual dots) against positions of involved nucleotides on the *x* and *y* axes (Fig. 1C). Any pairing interactions distanced by more than 400 nt were removed after analysis. By excluding long-distance interactions after whole-genome RNA structure prediction, we did not ignore the effect they may have on formation of local pairings. RNAalifold cannot predict pseudoknots, and therefore, the region directly downstream of the poly(C) tract was excluded from our analyses (Fig. 1). The presence of each conserved stem-loop was then further verified in each FMDV genomic sequence tested (Table S2).

10.1128/mSphere.00015-21.9 TABLE S2 List of the conserved stem-loops located in the ORF of the FMDV genome. Download Table S2, PDF file, 0.04 MB. © Crown copyright 2021. 2021 Crown https://creativecommons.org/licenses/by/4.0/ This content is distributed under the terms of the Creative Commons Attribution 4.0 International license.

These analyses correctly predicted the presence of well-characterized RNA secondary structures in the FMDV genome: the S-fragment, IRES, and *cre*, all located in the 5′ UTR, and SL1 and SL2 located in the 3′ UTR (Fig. 1C; see also Fig. S1 in the supplemental material). It additionally identified several serotype-specific conserved stem-loops in the region encoding the capsid proteins, but only four of these were conserved in all seven serotypes (Table S2). In contrast, 46 stem-loops (when counting each RNA hairpin individually, even within a single branched structure) were universally present within the regions encoding the nsps (Fig. 1C and Table S2). Overall, there were 53 highly conserved stem-loops in the ORF of the FMDV genome that were conserved across all seven serotypes, with average conservation in 95% of FMDV isolates tested (±7% SD [Table S2]).

### Use of CDLR mutagenesis for functional mapping of predicted RNA structures.

Next, we undertook mutagenesis studies to investigate whether any of the conserved RNA structures identified in the FMDV genome play a functional role in viral replication. FMDV replicons lack the region encoding the capsid proteins but are replication competent, demonstrating that there are no RNA elements essential for translation or replication of viral RNA within the capsid-encoding region. Therefore, our investigation focused on structures located within the regions encoding the nsps of the replicon. Additionally, the effect on replication of changes incorporated into the replicon can be analyzed in real time through monitoring of fluorescence from an integrated green fluorescent protein (GFP) reporter gene that replaced the region encoding the capsid proteins (65).

**FIG 2:**
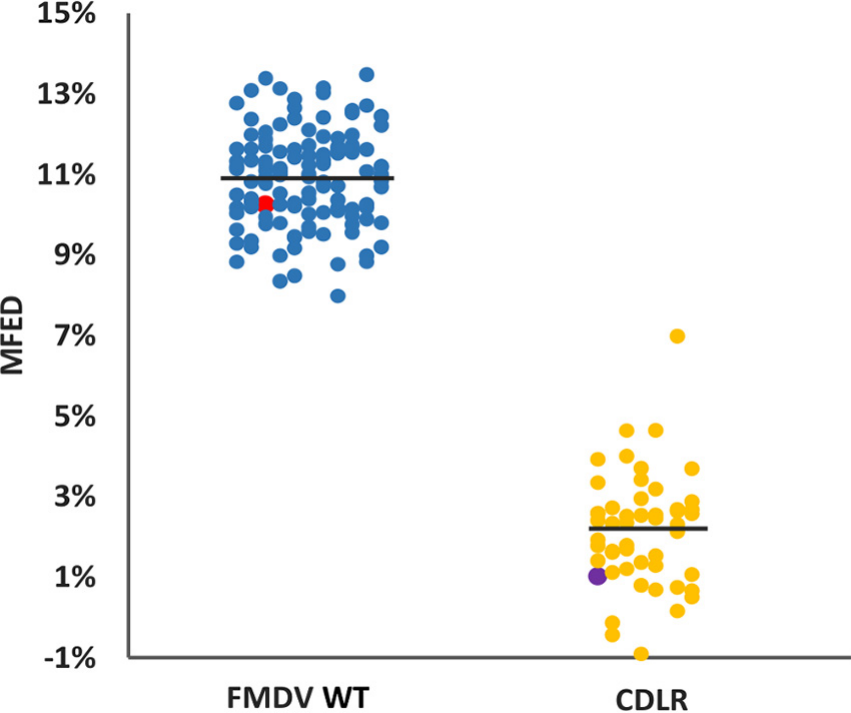
Comparison of average MFED values for wild-type (WT) and CDLR-scrambled sequences. Mean folding energy difference (MFED) for the regions encoding nonstructural proteins (nsps) of 118 FMDV field isolates representing all seven serotypes (blue dots), WT ptGFP replicon used in this study (red dot), and CDLR scrambled sequences (yellow dots). Among the latter is the CDLR scrambled sequence used in this study to generate replicon mutants (purple dot). To obtain CDLR-scrabbled sequences, the sequence of the regions encoding the nsps of the WT replicon was permuted 50 times by codon shuffling to minimize RNA secondary structure, while preserving protein coding, native dinucleotide frequencies, and codon usage.

In order to mutate the conserved RNA structures predicted within the regions encoding the nsps while maintaining codon composition, codon order, and dinucleotide frequencies of the native wild-type (WT) replicon sequence, we applied the CDLR scrambling method (11, 56). To monitor its effectiveness in altering or otherwise disrupting RNA pairing within the native sequence, sequence of the regions encoding the nsps of WT replicon was randomly permuted 50 times using the CDLR algorithm. Then, MFED values for these mutants were calculated as described above, and these values were compared to MFED values of the native WT replicon sequence and the corresponding sequences of the 118 FMDV isolates used in this study. Sequences generated by CDLR showed evidence of severely disrupted RNA secondary structures, with a mean MFED value of 2.2% (SD, ±1.4%), compared to a mean value of 10.9% (SD, ±1.2%) for the corresponding regions of the native FMDV sequences and that of the WT FMDV replicon (Fig. 2).

**FIG 3:**
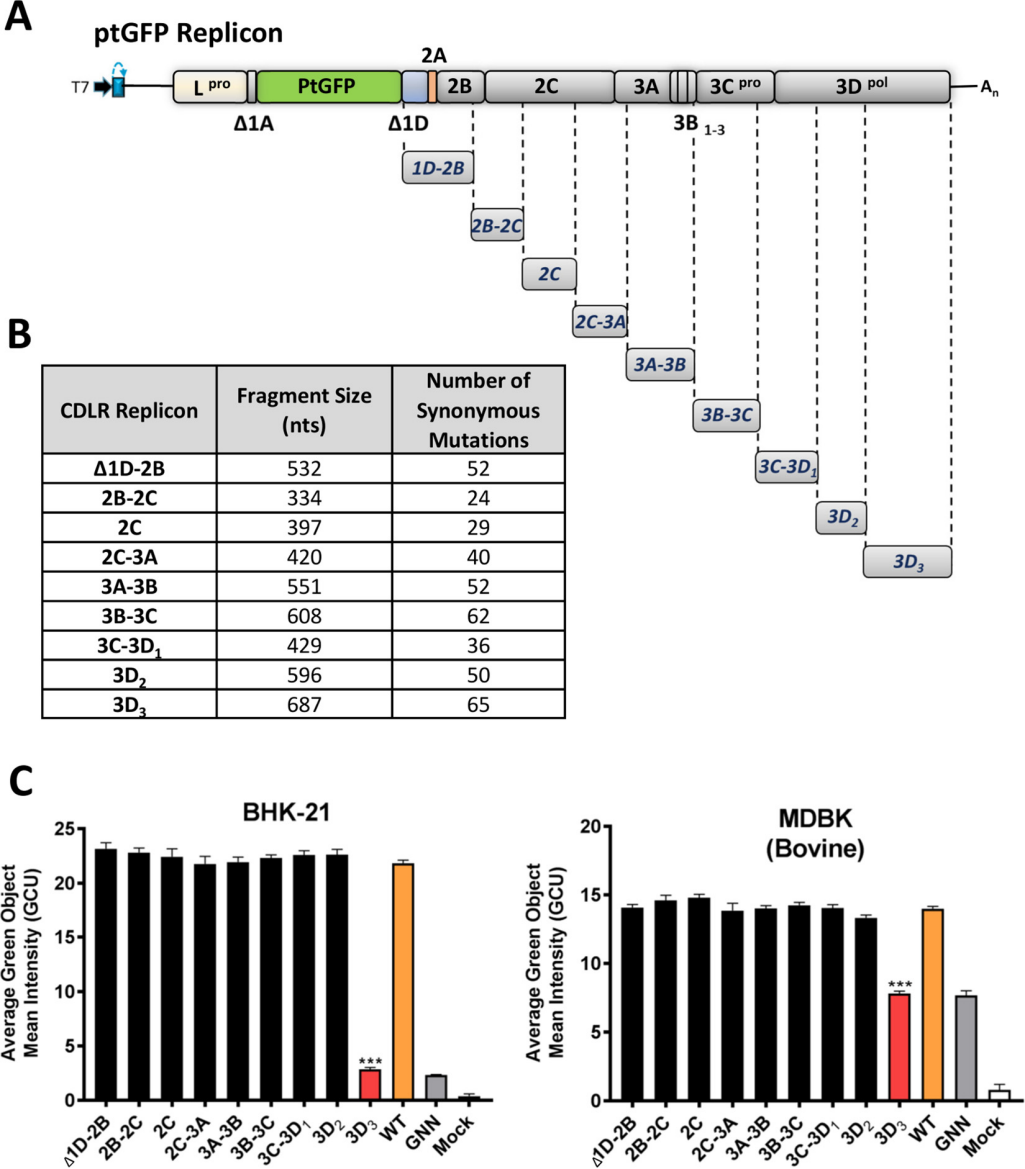
Replication of CDLR replicons within BHK-21 and MDBK cells. (A) Schematic representation of CDLR replicons. CDLR-permuted sequences were first inserted into a subclone encoding the nonstructural proteins (nsps) of the genome (Δ1D-polyA) before cloning into the WT ptGFP replicon. (B) CDLR replicon insert sizes and number of mutations within each region. Regions were chosen based on unique restriction site usage within the regions encoding nsps. Mutations were introduced as described in Materials and Methods. (C) IncuCyte data represent the average cell (green object) GFP intensity (also called green calibrated unit [GCU]) per well at 8 h posttransfection. Results are the means of three independent experiments plus standard errors (error bars). Significant differences between WT ptGFP and CDLR replicons were determined (***, *P* < 0.001). The replication-incompetent 3D^pol^ active site mutant (GDD → GNN) ptGFP-3D^pol^GNN was used as a negative control.

To identify functional RNA structures, we divided the regions encoding the nsps of the WT replicon (ptGFP replicon) into nine consecutive fragments defined by unique restriction sites and individually permuted each fragment using the CDLR algorithm (Fig. 3A and B). To further verify the extent of changes to the RNA structure introduced by the CDLR algorithm, we used the RNAforester program implemented in the ViennaRNA Package (64, 66, 67). This compared the putative structures adopted by the CDLR-permuted regions (Fig. 3A and B) to the structures located within the corresponding regions of the WT replicon sequence. RNAforester calculates RNA secondary structure alignments based on the tree alignment model and quantifies similarity of structures in question, where the relative similarity score values equal to one represent two identical structures (64, 66, 67). With the exception of the 2C-encoding region, which exhibits some structure similarity between CDLR and WT replicon (Fig. S2), there was low structural similarity between equivalent WT and CDLR genomic fragments (Table 1). RNA structures located in the 5′ and 3′ UTRs were generally unaffected by any CDLR permutation of the adjacent or more distal regions encoding the nsps, with the exception of the SL1 stem-loop in the 3′ UTR that was shorter by 11 pairings (Fig. S3).

**TABLE 1:** Similarity comparison of RNA structures within corresponding WT and CDLR replicon genomic fragments, calculated using RNAforester program

Comparison	Relative similarity score comparing RNA structure within replicon fragments^*a*^												
	Δ1D-2B	2B-2C	2C	2C-3A	3A-3B	3B-3C	3C-3D_1_	3D_2_	3D_3_	S-fragment	*cre* ^*b*^	IRES^*b*^	SL1 and SL2^*b*^
WT vs CDLR	−1.33	−0.71	0.24	−0.87	−0.80	−1.69	−1.06	−0.72	−1.66	1	1	1	0.64

a Relative similarity score comparing RNA structure located within nsps-encoding region fragments (as presented in Fig. 3A and B) of WT and CDLR replicon. A value of 1 is for two identical structures: the greater the distance from 1, the less structure similarity between two corresponding fragments. For simplicity, the output of RNAforester was rounded up to two decimal places.

b Comparison of RNA structures within the 5′ and 3′ UTRs of the WT and CDLR replicon acts as a control (note that while UTR regions were not permuted in this study, there was a possibility that permutation of the regions encoding the nsps might affect the pairings within UTRs).

10.1128/mSphere.00015-21.2 FIG S2 RNA structure for the whole genome of the WT replicon and a replicon with the 2C-encoding region permuted by the CDLR algorithm was predicted using RNAfold. The predicted structure of the 2C-encoding fragment was visualized using the Forna web service. The lines represent unstructured regions, and the numbers at the beginning and end of each individual stem-loop correspond to the genomic positions of each replicon. Nucleotides forming stems are shown in green, interior loops are shown in yellow, junctions in red, and hairpin loops in blue. Download FIG S2, PDF file, 0.4 MB. © Crown copyright 2021. 2021 Crown https://creativecommons.org/licenses/by/4.0/ This content is distributed under the terms of the Creative Commons Attribution 4.0 International license.

10.1128/mSphere.00015-21.3 FIG S3 RNA structure for the whole genome of the WT replicon and a replicon with the Δ1D-3D_3_-encoding region permuted by the CDLR algorithm was predicted using RNAfold. The predicted structures of SL1 and SL2 stem-loops located in the 3′ UTR were visualized using the Forna web service. The numbers correspond to the genomic positions of each replicon. Nucleotides forming stems are shown in green, interior loops are shown in yellow, hairpin loops in blue, and the unpaired regions in orange. Download FIG S3, PDF file, 0.3 MB. © Crown copyright 2021. 2021 Crown https://creativecommons.org/licenses/by/4.0/ This content is distributed under the terms of the Creative Commons Attribution 4.0 International license.

### CDLR replicon mutants reveal regions of secondary structure required for replication of an FMDV replicon.

Next, we examined the effect of RNA structure disruption on replication of the FMDV replicon using mutant replicons containing CDLR-permuted sequences over different parts of the regions encoding the nsps. For this, we used two different continuous cell lines known to support FMDV replication: baby hamster kidney (BHK)-21 cells routinely used to grow FMDV for vaccine production and Madin-Darby bovine kidney (MDBK) cells which originate from a natural host of FMDV and appear to have functional antiviral responses (68) (Fig. 3C). The replication kinetics of the mutant replicons was compared to the WT ptGFP replicon and a replicon with an inactive polymerase (ptGFP-3D^pol^GNN, previously described in reference 69). Since replication levels at 8 h posttransfection (hpt) were representative of the entire experiment (Fig. S4), for simplicity, data for this time point are shown. In both cell lines, all of the CDLR mutant replicons tested displayed replication kinetics comparable to the WT ptGFP replicon except for the replicon which carried a mutated sequence within the 3′-terminal part of the 3D^pol^-encoding region (called 3D_3_ here [Fig. 3C]). The replicon with mutated 3D_3_-encoding region was replication defective in both cell lines, with replication levels equivalent to the negative-control replicon (ptGFP-3D^pol^GNN; Fig. 3C). These results strongly suggest that this part of the 3D^pol^-encoding region contains RNA structures crucial for replication of the FMDV replicon. Consistent with the inferred location of the essential RNA structures in 3D_3_, CDLR permutation of the entire Δ1D-3A- and 3A-3D_2_-encoding region showed little effect on the replication kinetics (Fig. S5).

10.1128/mSphere.00015-21.4 FIG S4 Replication kinetics of FMDV replicon constructs containing the CDLR-permuted regions that were described in the legend to Fig. 3. IncuCyte data represent the average cell (green object) GFP intensity per well over a period of 12 h. Replicon RNA from CDLR mutants (black [red for 3D_3_]), WT ptGFP (orange), or ptGFP-GNN (gray) was introduced into BHK-21 or MDBK cell monolayers. Results are the means of three independent experiments ± standard errors (error bars). Download FIG S4, PDF file, 0.2 MB. © Crown copyright 2021. 2021 Crown https://creativecommons.org/licenses/by/4.0/ This content is distributed under the terms of the Creative Commons Attribution 4.0 International license.

10.1128/mSphere.00015-21.5 FIG S5 Replication kinetics of FMDV replicons containing larger sections of the region permuted by the CDLR algorithm. (A) Schematic representation of CDLR replicons encoding larger mutated regions. Mutated inserts were cloned directly into the ptGFP replicon using the unique restriction enzymes shown. (B) IncuCyte data represent the average cell (green object) GFP intensity per well over a period of 12 h within BHK-21 cells and MDBK cells. Results are the means of three independent experiments ± standard errors. Download FIG S5, PDF file, 0.3 MB. © Crown copyright 2021. 2021 Crown https://creativecommons.org/licenses/by/4.0/ This content is distributed under the terms of the Creative Commons Attribution 4.0 International license.

### Modification of individual stem-loops within the 3D_3_ region impairs replication of an FMDV replicon.

**FIG 4:**
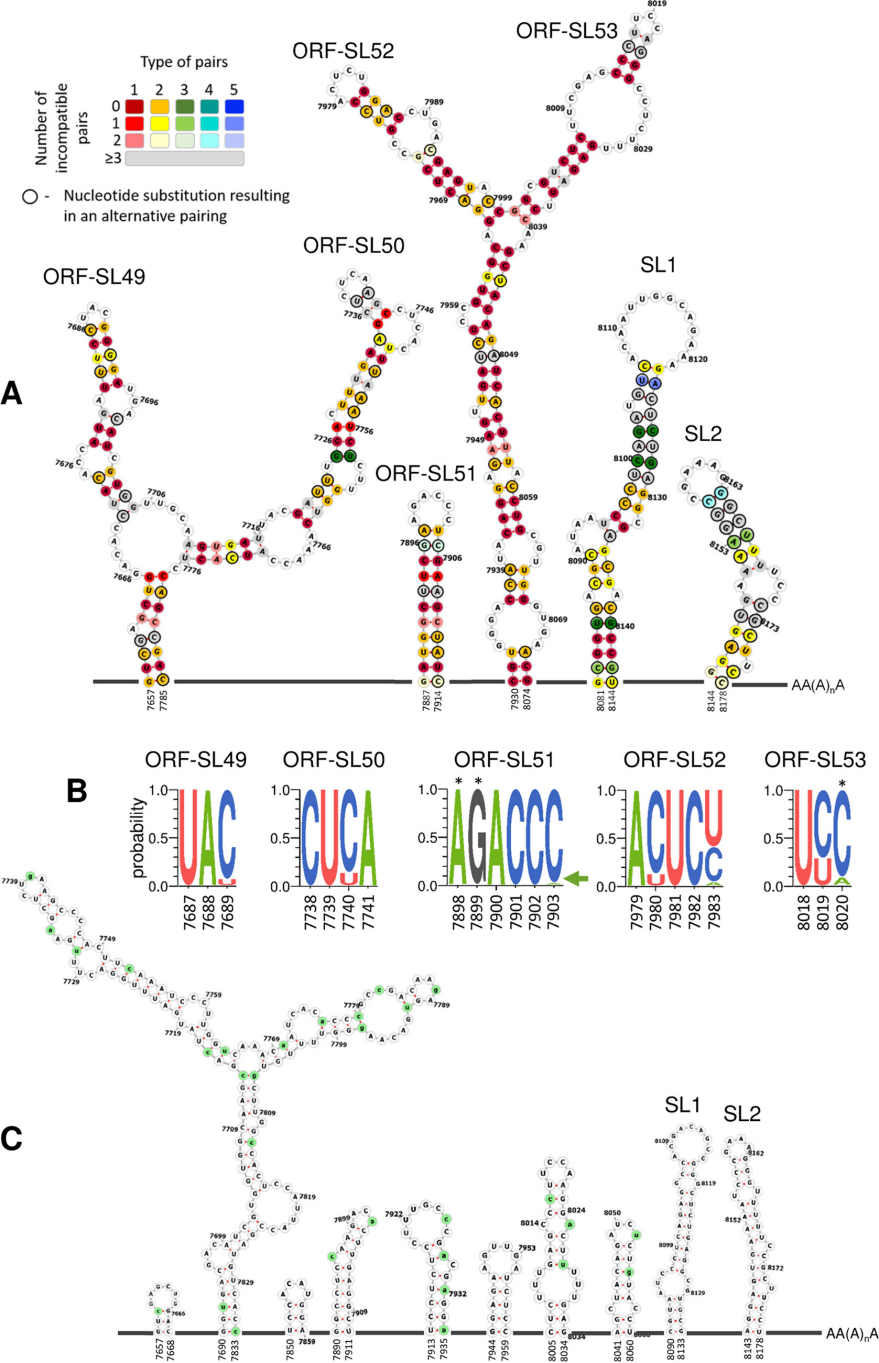
Schematic representation of predicted conserved RNA structures located at the 3′-terminal end of the region encoding 3D^pol^. (A) Schematic representation of conserved (in all FMDV serotypes) RNA secondary structures located at the 3′-terminal end of the region encoding 3D^pol^ (i.e., 3D_3_). Conserved putative stem-loops (ORF-SL49 to ORF-SL53) are shown, where two stem-loops located in the 3′ UTR described before (SL1 and SL2) act as the control of the computational prediction. Nucleotide positions which form conserved pairing were color coded according to number of pairing types (red for 1 and blue for 5) and conservation of a pairing (dark shades for nucleotide pairing occurred in all FMDV isolates to light shades for lack of nucleotide pairing in two FMDV isolates). Positions colored in light gray show lack of pairing for three or more FMDV isolates. Black circular outline indicates nucleotide position where a substitution resulted in an alternative pairing (see the legend in the top left corner of panel A for detail). Unstructured regions are represented as dark gray lines and are not drawn to scale. Table S3 in the supplemental material specifies details represented graphically in the color legend in the top left corner of panel A. (B) Extent of nucleotide conservation within hairpin loops of ORF-SL49 to ORF-SL53 RNA structures. Sequence logos were generated using WebLogo 3.7.4 web server based on sequences of 118 FMDV isolates. Probability shows the extent of nucleotide occurrence at a given position. Asterisks mark positions where substitution occurs in 1 out of 118 FMDV isolates, but due to a limited resolution of the *y* axis, it does not appear in the sequence logos (these are A7898G, G7899A, and C8020G). The green arrow points to the C7903A substitution which due to the height of the A symbol could go unnoticed. (C) Schematic representation of RNA secondary structures located in the 3D_3_ region after scrambling using the CDLR algorithm, demonstrating how RNA secondary structure in this region was changed. Mutated nucleotide positions are highlighted in green. Unstructured regions are represented as dark gray lines and are not drawn to scale. For all three panels (A to C), numbers represent nucleotide positions corresponding to the sequence of A/Brazil/1979 isolate (GenBank accession number AY593788).

Our results indicate that the 3D_3_ region of the FMDV genome contains conserved secondary RNA structures that may be necessary for replication of the FMDV replicon. Therefore, the RNA structures present in this region were investigated in more detail by visualizing each individual structure and comparing it to the corresponding scrambled region within the CDLR mutant. Analysis of corresponding sequences of FMDV field isolates (over the 3D_3_ region) revealed five stem-loops (ORF-SL49 to ORF-SL53) with strong nucleotide pairing conservation, with ORF-SL52 being the most conserved structure (Fig. 4A). Variability within all structures was accommodated though the occurrence of covariant changes that preserved nucleotide pairings (Fig. 4A). Additionally, there was substantial nucleotide sequence conservation in the sequence forming the unpaired loop at the top of the stem-loop structures (i.e., in the hairpin loops) of ORF-SL49, ORF-SL50, and ORF-SL51 (Fig. 4B), implying some functional constraints on these sequences. Each of the predicted structures in the WT sequence was substantially disrupted in the CDLR scrambled mutant (Fig. 4C).

10.1128/mSphere.00015-21.10 TABLE S3 Supplemental legend for Fig. 4A. Data in this table show the values depicted in the color legend in the top left corner of Fig. 4A. Download Table S3, PDF file, 0.04 MB. © Crown copyright 2021. 2021 Crown https://creativecommons.org/licenses/by/4.0/ This content is distributed under the terms of the Creative Commons Attribution 4.0 International license.

**FIG 5:**
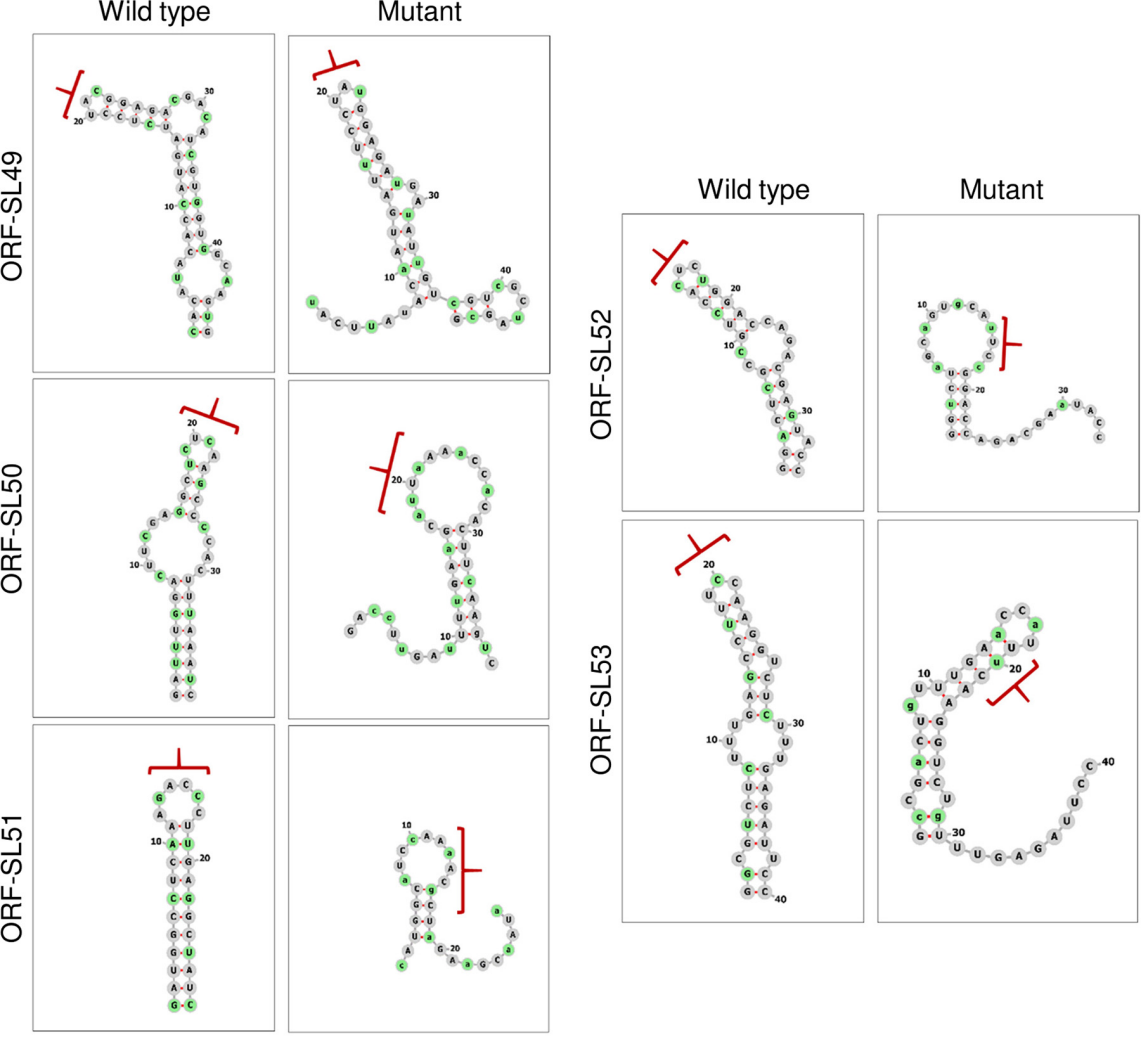
Disruption of the predicted RNA secondary structures by silent mutagenesis. The conserved stem-loops identified in the 3′-terminal end of the region encoding 3D^pol^ (i.e., 3D_3_) of FMDV were predicted individually by Mfold for the WT ptGFP replicon. Predicted WT stem-loops were mutated to cause the highest possible disruption or change to the RNA structure without affecting neighboring stem-loops while keeping the same amino acid sequence and dinucleotide ratio (i.e., CpG and UpA). Predicted WT and mutated stem-loops visualized using the Forna web server are shown. Nucleotides highlighted in green represent mutated positions, while red brackets represent positions of the hairpin loop in the WT structures and their altered position in the disrupted structures after mutagenesis.

**FIG 6:**
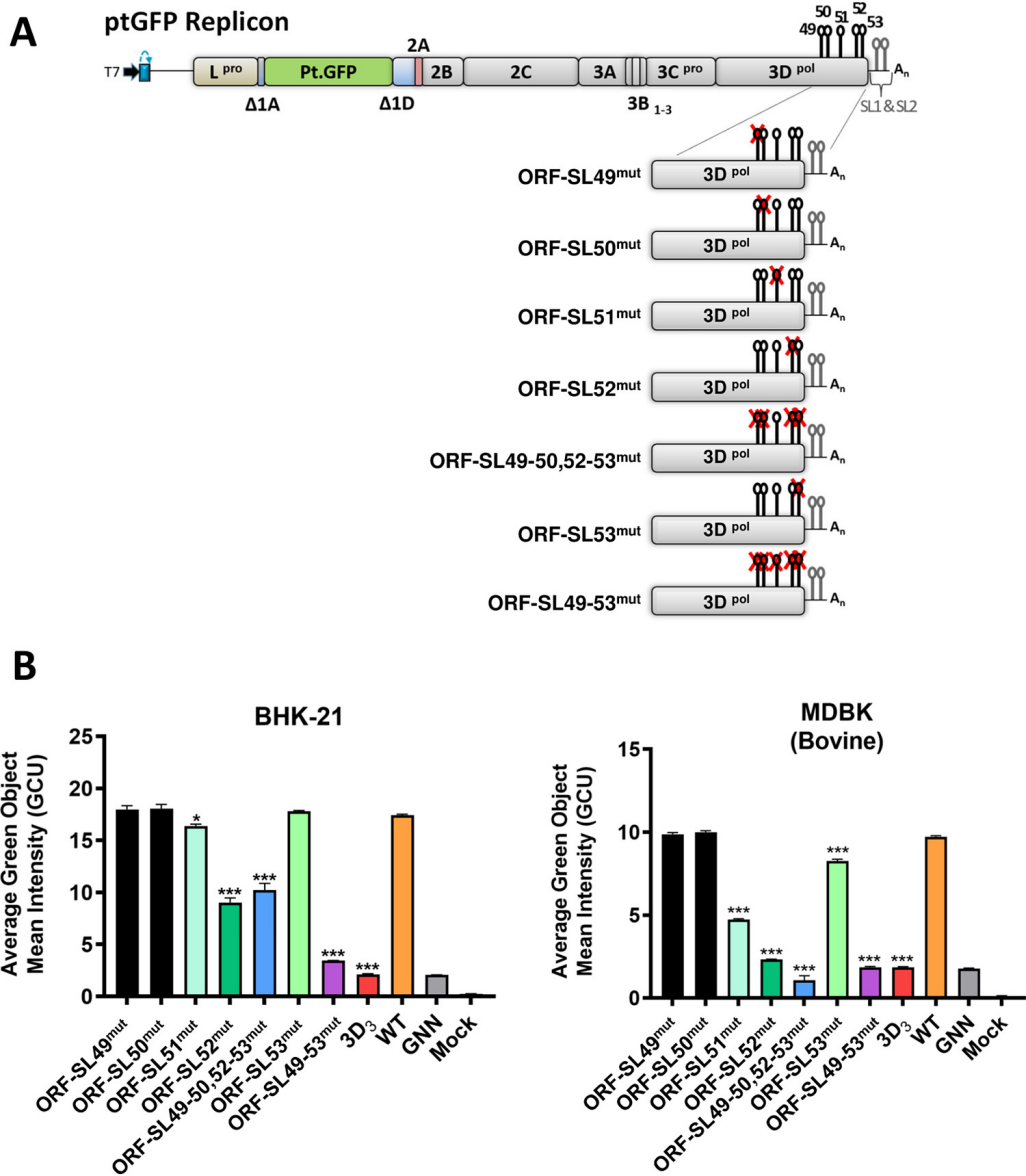
Effect of individual stem-loop (ORF-SL49, -50, -51, -52, and -53) mutagenesis on replication of the FMDV replicon. (A) Schematic representation of FMDV replicon constructs containing stem-loop mutations (individually, ORF-SL49^mut^, ORF-SL50^mut^, ORF-SL51^mut^, ORF-SL52^mut^, and ORF-SL53^mut^; in combination, ORF-SL49-50,52-53^mut^). Please see Results for detailed description of mutants. Sequence inserts containing stem-loop mutations were cloned directly into the ptGFP replicon. (B) IncuCyte data represent the average cell (green object) GFP intensity per well at 8 h posttransfection within BHK-21 and MDBK cells. Results are the means of three independent experiments plus standard errors. Significant differences between WT ptGFP and ORF-SL^mut^ replicons were determined (*, *P* < 0.05; ***, *P* < 0.001).

Further studies were therefore undertaken to dissect the importance of the individual stem-loops within the 3D_3_ fragment for replication of the FMDV replicon. Each of the five putative RNA structures in the 3D_3_ region of the WT replicon was permuted individually *in silico*, introducing the maximum number of nucleotide changes possible to disrupt the RNA structure while maintaining amino acid coding, dinucleotide frequencies, and the integrity of the neighboring RNA structures (Fig. 5 and 6A). Additionally, a replicon where all five putative RNA stem-loops were altered (ORF SL49-53^mut^, using the same mutation strategy as for each individual stem-loop [Fig. 6A]) was designed to confirm that mutation of these particular stem-loops, and not of other elements present in the CDLR replicon with the permuted 3D_3_ region, impaired RNA replication. Replication of ptGFP replicons carrying individual mutated stem-loops was tested in the same two cell lines as described above (Fig. 6). As previously observed, replication levels at 8 hpt were representative of the replication kinetics (Fig. S6). Replication of replicons with disrupted ORF-SL49 and ORF-SL50 was not affected in either cell line (Fig. 6B and Table 2). In contrast, replication of replicons with disrupted ORF-SL51 or ORF-SL52 was significantly reduced, although the effect on replication varied between the cell lines. Disruption of ORF-SL51 led to only a marginal, but statistically significant, reduction of replication in BHK-21 cells, whereas the negative effect on replication in MDBK cells was greater (Fig. 6B and Table 2). In both cell lines, disruption of ORF-SL52 reduced replication to a greater extent than disruption of ORF-SL51, with the replication profile in bovine cells being close to the replicon with an inactive polymerase (ptGFP-3D^pol^GNN) and the replicon with the 3D_3_ region mutated by the CDLR algorithm (Fig. 6B and Table 2). Replication of the replicon with disrupted ORF-SL53 was reduced only in MDBK cells (Fig. 6B and Table 2). Finally, the replicon with all five stem-loops altered (ORF SL49-53^mut^) demonstrated replication comparable to the ptGFP-3D^pol^GNN replication-deficient control (which gives a GFP signal due to translation of the input RNA [Fig. 6B and Table 2]).

**TABLE 2:** Summary of replication profiles of FMDV replicons after mutagenesis of conserved stem-loops localized within the 3D_3_ genomic region

Cell line	Replicon profile^*a*^										
	ORF-SL49^mut^	ORF-SL50^mut^	ORF-SL51^mut^	ORF-SL52^mut^	ORF-SL53^mut^	ORF-SL49-50, 51-52^mut^	ORF-SL51,52^mut^	ORF-SL51,53^mut^	ORF-SL51-53^mut^	ORF-SL49-53^mut^	GNN^*b*^
BHK-21	WT	WT	94%*	52%	WT	59%	27%	60%	28%	23%	11%
MDBK	WT	WT	49%	24%	85%	11%	20%	36%	20%	20%	18%

a See Fig. 6 and 7 and their legends for study design and data. WT, wild-type replicon-like replication profile. Values with percentage symbols show the percentage of the WT ptGFP signal, where a significant effect on replicon replication was observed (*P* value < 0.001), except for ORF-SL51^mut^ in BHK-21 cells where *P* value = 0.02, indicated by the asterisk.

b GNN is a replicon with an inactive polymerase. Any GFP signal is due to translation.

10.1128/mSphere.00015-21.6 FIG S6 Replication kinetics of FMDV replicon constructs containing the individual stem-loop mutants that were described in the legend to Fig. 6. IncuCyte data represent the average cell (green object) GFP intensity per well over a period of 12 h within BHK-21 cells and MDBK cells. Results are the means of three independent experiments ± standard errors. Download FIG S6, PDF file, 0.2 MB. © Crown copyright 2021. 2021 Crown https://creativecommons.org/licenses/by/4.0/ This content is distributed under the terms of the Creative Commons Attribution 4.0 International license.

The effect of the combined mutagenesis of stem-loops on replication of the FMDV replicon was also investigated. Initially, we tested a replicon with those four stem-loops (ORF-SL49, ORF-SL50, ORF-SL52, and ORF-SL53) mutated (ORF-SL49-50,52-53^mut^), which have not been predicted before. Since mutation of ORF-SL49 and ORF-SL50 had no apparent effect on replicon replication, this replicon showed the effect of combined mutagenesis of the ORF-SL52 and ORF-SL53. While in BHK-21 cells ORF-SL49-50,52-53^mut^ replicon was attenuated at a level similar to that of the replicon containing mutation of the ORF-SL52, in the MDBK cells ORF-SL49-50,52-53^mut^ replicon showed replication levels even lower than the replicon with an inactive polymerase (Fig. 6B and Table 2), suggesting that combined mutation of the ORF-SL52 and ORF-SL53 might affect translation of the viral genome. Further constructs with two loops disrupted (ORF-SL51,52^mut^ and ORF-SL51,53^mut^), or all three loops disrupted (ORF-SL51-53^mut^) were tested (Fig. 7). In both cell lines, disruption of ORF-SL51 in combination with ORF-SL52 (ORF-SL51,52^mut^), or in combination with ORF-SL52 and ORF-SL53 (ORF-SL51-53^mut^) resulted in replication levels comparable to that of the ORF-SL49-53^mut^ negative control (Fig. 7B and Table 2). In BHK-21 cells, replication of the ORF-SL51,52^mut^ showed synergistic reduction compared to replicons with the ORF-SL51 and ORF-SL52 mutated individually (Fig. 7B and Table 2). Interestingly, disruption of ORF-SL53 in combination with ORF-SL51 (ORF-SL51,53^mut^) resulted in a significant reduction of replicon replication in both cell lines (Table 2 and Fig. 7), although in BHK-21 cells individual mutation of ORF-SL51 and ORF-SL53 had only a marginal effect or no effect, respectively (Fig. 6). Our computational prediction of ORF-SL51,53^mut^ did not suggest any disruption of the ORF-SL52 secondary structure, which was indirectly confirmed by the experimental data where replication impairment caused by joint permutation within ORF-SL51,53^mut^ was significantly less than that of the ORF-SL51-53^mut^ (Table 2 and Fig. 7B).

**FIG 7:**
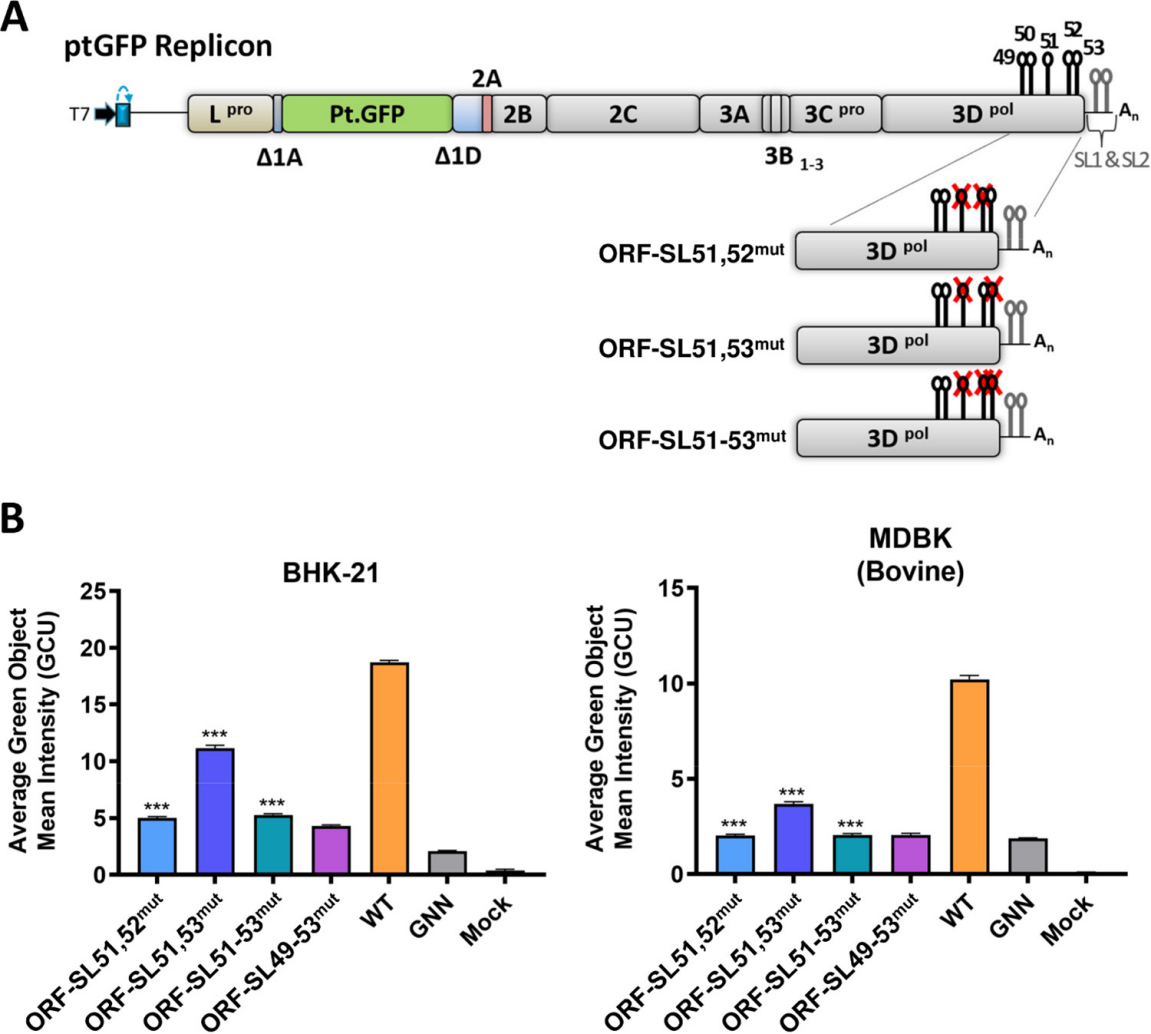
Effect of combined mutagenesis of stem-loops ORF-SL51, ORF-SL52, and ORF-SL53 on replication of the FMDV replicon. (A) Schematic representation of FMDV replicon constructs containing combined stem-loop mutations (ORF-SL51,52^mut^, ORF-SL51,53^mut^, and ORF-SL51-53^mut^). Sequence inserts containing stem-loop mutations were cloned directly into the ptGFP replicon. (B) IncuCyte data represent the average cell (green object) GFP intensity per well at 8 h posttransfection. Results are the means of three independent experiments plus standard errors. Significant differences between WT ptGFP and ORF-SL^mut^ replicons were determined (***, *P* < 0.001).

### Comparison of the conserved stem-loops within the FMDV 3D_3_ region to structures found in the 3′-terminal 3D^pol^-encoding region of poliovirus.

Two stem-loops (referred to as loop α and β by Song et al. [38]) necessary for poliovirus (PV) replication are present in the 3′-terminus-encoding sequence of PV 3D^pol^ (37). Since PV is a member of a different genus in the family *Picornaviridae* and distantly related to FMDV, we investigated whether any of the stem-loop structures found in the 3′ end of the 3D^pol^-encoding region of the FMDV genome were similar to those present in the equivalent part of the PV genome. Therefore, we compared each of the FMDV RNA structures (ORF-SL49 to ORF-SL53) to the PV loops α and β using RNAforester. As described in Table 3, the structures identified in the 3′-terminal part of the coding region of FMDV 3D^pol^ do not appear to resemble those found in the equivalent position of the PV genome, while (using the same approach) the *cre* structures of PV and FMDV showed some structural similarity.

**TABLE 3:** Similarity comparison of RNA structures within the 3D^pol^-encoding region of FMDV and PV, calculated using RNAforester program

PV RNA structure	Similarity^*a*^ of the indicated RNA structures of FMDV and PV					
	ORF-SL49	ORF-SL50	ORF-SL51	ORF-SL52	ORF-SL53	*cre* ^*b*^
Loop α	−1.46	−1.83	−2.30	−2.10	−1.85	ND
Loop β	−1.18	−2.07	−2.52	−2.27	−2.07	ND
*cre* ^*b*^	ND	ND	ND	ND	ND	0.30

a A relative similarity score of 1 indicates two identical structures: the greater the distance from 1, the less structure similarity between the two compared features. For simplicity, the output of RNAforester was rounded up to two decimal places. ND, not determined.

b Comparing PV *cre* to FMDV *cre* acts as a control of the structure prediction and RNAforester analysis.

## DISCUSSION

Many aspects of FMDV replication remain poorly understood, such as the function of RNA structures found within the ORF. Here, we revisited the RNA structural architecture of the FMDV genome and, for the first time, investigated whether the putative stem-loops localized within the ORF are required for viral genome replication. Our results are in line with previous studies showing that FMDV has extensive RNA structure throughout the genome, substantially exceeding that found in viruses of other genera of the family *Picornaviridae* (e.g., MFED value of >10% for FMDV genomic sequences compared to <4% for viral sequences belonging to genera *Enterovirus*, *Hepatovirus*, *Parechovirus*, and *Teschovirus*) (12, 56, 62). Compared to the previous structure predictions performed by Witwer et al. (12), our study identified a greater number of conserved RNA structures within the FMDV ORF (53 stem-loops, with some merging into 46 branched structures, versus 25 structures predicted previously). Since we used a larger data set than the previous authors (118 relatively diverse FMDV sequences versus 9 used by Witwer et al. [12]), it is possible that we obtained a stronger statistical signal supporting conservation of these additional structures. Importantly, we found that three of the structures within the coding region of 3D^pol^ (i.e., ORF-SL51, ORF-SL52, and ORF-SL53) were critical for efficient replication of an FMDV replicon, thereby implying that they would provide the same function during virus replication.

Despite consistently elevated MFED values, the FMDV capsid-encoding region contained only four RNA stem-loops which were conserved in all serotypes. Viral genomes characterized by high MFED values and low conservation of individual RNA structures have been observed before (70). For instance, the coding region of hepatitis C virus (HCV) showed elevated MFED values, while, except for the terminal genomic regions, the individual stem-loop structures were distinct between different HCV genotypes and even subtypes (70–72). Similarly, FMDV showed dense serotype-specific RNA structure within its capsid-encoding region, which were not shared among other serotypes (as found in reference 70 and independently here).

To identify functional RNA structures, we applied the CDLR algorithm to permute a genomic FMDV sequence (63). While the degree of possible mutagenesis is necessarily limited by protein coding, dinucleotide frequency, and codon usage constraints, the CDLR algorithm substantially disrupted secondary RNA structure of the native FMDV sequence in all regions apart from the region encoding 2C (Table 1 and Fig. S2). Since the permutation of the entire Δ1D-3A-encoding region (which resulted in more extensive changes to the RNA structure) had a minimal effect on replication of the FMDV replicon, it is safe to state that conserved RNA stem-loops within the 2C-encoding region are not essential for replication of the FMDV replicon *in vitro*.

In contrast, the CDLR scanning method identified three structures located at the 3′-terminal part of the 3D^pol^-encoding region that were important for replication of the FMDV replicon. Of these structures, ORF-SL52 showed the highest degree of pairing conservation and appeared to be the predominant structure important for replication of the FMDV replicon. Mutation of ORF-SL51, ORF-SL52, or ORF-SL53 showed a much greater reduction of replicon replication in MDBK cells compared to BHK cells. MDBK cells have been shown to secrete high levels of interferon (IFN) upon stimulation (68), while BHK-21 cells are known to lack an intact IFN pathway (73, 74). Furthermore, several published results suggest that RNA structure might directly or indirectly play a role in the modulation of antiviral responses (42, 54, 75, 76). Collectively, these observations suggest that ORF-SL51, ORF-SL52, and ORF-SL53 could play additional roles in the evasion of antiviral responses, and therefore, mutation of these structures led to a drastic reduction in replication of the FMDV replicon in IFN-competent cell lines. In both cell lines tested, deletion of two or more stem-loops (ORF-SL51, ORF-SL52, and/or ORF-SL53) in combination significantly impaired replication of the replicon, suggesting that even in the absence of a fully functional antiviral pathway, all three stem-loops are important for FMDV replication. While the mechanism of stem-loop involvement in FMDV replication remains unknown, the synergistic decrease in replication of the FMDV replicon upon deletion of ORF-SL51,53 or ORF-SL52,53 in BHK-21 cells may suggest involvement of RNA-RNA interactions. Similarly to the viral genome, replication of an FMDV replicon involves viral protein synthesis, and the sequential synthesis of negative-strand (i.e., complementary) and positive-strand (i.e., genomic) viral RNA. Thus, although ORF-SL51-53 are required for replication of the replicon, further studies are required to dissect which of these processes (viral RNA translation and/or viral RNA replication) are dependent on ORF-SL51, ORF-SL52, and ORF-SL53. Interestingly, in the PV genome, stem-loops within the coding region of 3D^pol^ have been identified that are required for viral RNA synthesis (37, 38). However, these structures do not appear to share sequence or structural similarity with ORF-SL51, ORF-SL52, or ORF-SL53 in the FMDV genome.

The observation that replication of the FMDV replicon mutants with disrupted RNA structure elsewhere in the regions encoding the nsps (i.e., spanning 1D through to most of 3D^pol^) was surprising. The maintenance of extensive conserved internal base pairing and consistently elevated MFED values observed in the relatively diverse set of FMDV isolate sequences analyzed indeed strongly argues that the RNA structures formed by those genomic regions must play some functional role in the FMDV replication cycle. It is possible that at least some of the apparently “nonfunctional” RNA structures are genome-scale ordered RNA structure (GORS) which may play a role in persistence of FMDV in its natural host (56, 62). While FMDV causes an acute disease in domestic animals (14, 77), it is known to persist in African buffalo (Syncerus caffer), which are a natural reservoir of the virus (78–81). Since FMDV and African buffalo are thought to have coevolved together, it is possible that GORS developed in the FMDV genome as a part of the virus-host coadaptation, where they might assist in evasion of immune recognition. The link between GORS, persistence, and ability to minimize antiviral sensing has been shown for a number of unrelated viruses (56, 62, 70, 75). Work is under way to investigate whether any of these remaining structures play a role in modulation of the antiviral sensing during FMDV replication in its natural host environment.

Although the function of the apparently nonessential RNA structures within the regions encoding the nsps remains to be defined, due to their conserved nature, they form a potential target for genome-scale attenuation of a wide range of FMDV strains. Such a strategy could contribute to the development of live attenuated FMD vaccines that may improve on the short duration of immunity, which is a shortcoming of current inactivated vaccines. Alternatively, the manipulation of RNA structures such as ORF-SL51 to provide attenuation in bovine cells but retain efficient growth in vaccine production cell lines (BHK) could be used to enhance biosafety of inactivated vaccine production. The hazards associated with the large-scale production of killed vaccine viruses include both accidental release of virus from high containment production facilities, and the distribution and use of improperly inactivated FMD vaccines (82–84).

In summary, we have generated a comprehensive map of RNA secondary structure located within the ORF of the FMDV genome and identified novel stem-loops within the coding region for 3D^pol^ that appear critical for FMDV replication. While the function of the other conserved structures remains to be determined, they can be targeted to improve understanding of the FMDV biology. In addition, they have the potential to help develop safer FMDV vaccines, an idea which has been proposed for other viruses (6, 56, 85). We also show that usage of the CDLR algorithm can be successfully utilized to permute RNA sequences in search of functional RNA structures, which can be applied beyond viral RNA molecules using a freely available and easy to use package (63).

## MATERIALS AND METHODS

### Cell lines.

Madin-Darby bovine kidney (MDBK) and baby hamster kidney (BHK-21) cells were obtained from the American Type Culture Collection (ATCC) and maintained in Dulbecco’s modified Eagle medium (DMEM) containing either 10% fetal bovine serum (FBS) or 10% horse serum (MDBK cells) at 37°C and 5% CO_2_.

### FMDV sequence data set.

Full genome sequences of 105 viruses were selected from GenBank database based on nucleotide distance of their 1D-encoding region, ensuring that they represent sequence variability that is known to be present between all seven FMDV serotypes (see Table S1 in the supplemental material). The 1D-encoding region is the most variable part of the FMDV genome and therefore was used to calculate distance between candidate isolates. Since sequences of SAT serotypes are the least represented on public databases, 13 additional full genome sequences of field SAT isolates (SAT 1 = 2, SAT 2 = 4 ,and SAT 3 = 7) were generated for the purpose of this study [isolates SAT1/TAN/3/80, SAT1/ZAM/2/88, SAT2/BOT-BUFF/7/72, SAT2/MOZ/1/70, SAT2/ZAM-BUFF/18/74, SAT2/ZIM/8/89, SAT3/BOT/209/67, SAT3/RHO/26/76, SAT3/RHO/3/75, SAT3/SAR/9/79, SAT3/ZAM/P2/96(MUL-4), SAT3/ZIM/P25/91(UR-7), and SAT3/ZIM/P26/90(HV-5)] using methodology previously described (45).

### Prediction of conserved RNA structures within the FMDV genome.

The genomic sequences of the 118 FMDV field isolates were aligned using the MAFFT X-INS-i algorithm which, in addition to nucleotide identity, takes into account RNA secondary structure information (86, 87). The multiple sequence alignment (MSA) was analyzed using the RNAalifold program implemented in the ViennaRNA package (64), using the following options: a ribosum scoring matrix, calculating the partition function and base pairing probability matrix in addition to the minimum free energy (MFE) structure, producing structure without lonely pairs and with dangling energies added for the bases adjacent to a helix on both sides. Then, the conserved RNA structures in the full genome were “tidied up” by removing gaps and long-distance interactions. The same was repeated for each FMDV serotype individually (using the data set described above), and serotype-specific conserved RNA structure prediction was compared to the conserved structure prediction for all 118 FMDV sequences. Stem-loops which were verified in all seven FMDV serotypes were visualized by drawing a dot plot graph using an awk script written in-house and available upon request. To visualize RNA structure located within shorter genomic fragments, a particular genomic region together with its conserved structure prediction was extracted and visualized using an on-line Forna tool implemented in the ViennaRNA Web Services (88). The extent of nucleotide conservation in sequence forming hairpin loops of RNA structures was visualized using a WebLogo 3.7.4 web server (89, 90).

Pairwise distance and MFED for full genome sequences were prepared using the Sequence Distances and Folding Energy Scan programs implemented in SSE v1.4 package (63), respectively. The MSA for MFED analysis was prepared as described above, while controls for calculation of MFED were generated by the NDR algorithm. For sequence distance analysis, the FMDV genomes were separated into three genomic regions: the 5′ UTR, ORF, and 3′ UTR which were aligned individually by different MAFFT algorithms. The 5′ and 3′ UTRs were aligned by MAFFT X-INS-i, while the nucleotide sequence of the ORF was first converted into amino acid sequence using TRANSEQ EMBOS program (91), aligned using MAFFT G-INS-i (92), and then such generated amino acid alignment was converted into nucleotide sequence using TRANALIGN EMBOS program (91). All aligned genomic fragments were manually combined into a single MSA containing FMDV whole genomes.

The average MFED values of the regions encoding the nsps of the FMDV isolates, the ptGFP replicon, and 50 CDLR-permuted ptGFP mutants were calculated as described above.

Since there appears to be a lot of ambiguity around the poly(C) tract, that region and its flanking positions were excluded from all analyses.

### *In silico* design of mutants containing modified segments within the nonstructural-protein-encoding region.

The regions encoding the nsps of the FMDV genome were chosen for mutagenesis by restriction site usage (Fig. 3A and B). To disrupt RNA secondary structures predicted in each restriction fragment of native FMDV genomes, sequences were mutated using the CDLR algorithm implemented in the Scramble Sequences Program of the SSE v1.4 package.

Structure prediction of the 3′-terminal part of the 3D^pol^-encoding region of the WT replicon which was scrambled by the CDLR algorithm (the 3D_3_ region) was generated as described above but using RNAfold (64).

To “quantify” the difference between the structures of the WT and scrambled replicons (Fig. 3A and B and Table 1), the whole genomic sequence of WT and each scrambled replicon was predicted using RNAfold, and fragments of the RNA secondary structure prediction corresponding to the permuted regions encoding the nsps (Fig. 3A and B) were compared using RNAforester and global alignment (64, 66, 67).

For each predicted RNA structure located at the 3′-terminal part of the FMDV 3D^pol^-encoding region (ORF-SL49 to ORF-SL53), nucleotides were changed manually. Individual putative stem-loops and their mutants were predicted using RNAfold implemented in the ViennaRNA package and mfold RNA structure prediction server (93) and were visualized using Forna.

### Comparison of putative RNA structures located within 3′-terminal 3D^pol^-encoding region of FMDV and PV.

Computational prediction of two conserved poliovirus (PV) RNA structures located in the 3′-terminal 3D^pol^-encoding region (termed loop α and β as in Song et al. [38]) and described previously (37, 38) was repeated as described above. This was done as there was some discrepancy between the two publications about the exact structure of the two PV stem-loops. PV sequences representing variability of the PV 3D-encoding region (GenBank accession numbers NC_002058.3, DQ890388.1, FJ769378.1, EU794963.1, AY560657.1, HF913427.1, EU794957.1, EU794956.1, AF538842.1, EU684057.1, AF405667.1, AF405666.1, KJ170457.1, KJ170438.1, KU866422.1, AM884184.1, AJ132961.1, MG212491.1, MG212488.1, MG212485.1, MG212463.1, MG212456.1, MG212441.1, MG212440.1, KY941933.1, KY941932.1, KR259355.1, KC784372.1, KC880377.1, JX275352.1, JX274995.1, and KX162704.1) were used. The RNA loops α and β were isolated, and their structures were aligned to the 3′-terminal part of the 3D^pol^-encoding region containing FMDV stem-loops ORF-SL49 to ORF-SL53 using the RNAforester software and “small-in-large similarity” calculation to determine whether any of the previously described PV stem-loops were similar to any of the FMDV RNA structures identified in this study. For more detailed analysis, each isolated FMDV putative RNA stem-loop (ORF-SL49 to ORF-SL53) was directly compared to PV loop α or β using RNAforester as described above.

### Clone construction.

Sequences with mutations generated by the CDLR algorithm were synthesized by custom DNA synthesis (GeneArt, Life Technologies) and provided within standard cloning vectors. Since the restriction sites flanking nine CDLR regions (Fig. 3B) were not unique, final replicons containing CDLR-permuted regions were generated using subclones which allowed for usage of convenient restriction sites.

The nucleotide fragments containing mutated stem-loops ORF-SL49 to ORF-SL53 were synthesized as described above and directly cloned into the ptGFP replicon.

### *In vitro* transcription.

Replicon constructs (5 μg) were linearized with AscI (New England Biolabs) for 1 h at 37°C and purified using the E.Z.N.A. gel extraction kit (Omega Bio-Tek). Linear replicon DNA (500 ng) was added to transcription reaction mixtures at a final volume of 100 μl containing the following: Transcription Optimized buffer (Promega), 10 mM dithiothreitol (DTT) (Promega), 100 U RNasin RNase inhibitor (Promega), 40 U T7 RNA polymerase (Promega), 20 mM ribonucleotide triphosphates (rNTPs) (Promega), and nuclease-free water. Reaction mixtures were incubated at 37°C for 2 h, and the resulting transcript integrity was assessed by agarose gel electrophoresis. RNA yield was quantified using the Quantus fluorometer (Promega), according to the manufacturer’s instructions.

### Cell transfection.

Approximately 20 h prior to transfection, cells were seeded into 24- or 12-well plates at the appropriate cell seeding density to achieve ∼80% confluence. The following day, medium was removed and replaced with FluoroBrite DMEM (Gibco) supplemented with 2% FBS and 4 mM glutamine. Replicon transcript RNA (0.5 to 1 μg) was transfected into triplicate or quadruplicate cell monolayers using Lipofectamine 2000 transfection reagent (Thermo Fisher Scientific) per the manufacturer’s recommendation.

### Live cell imaging.

Live cell image analysis was performed using the IncuCyte ZOOM kinetic imaging system (Essen BioScience) as described previously (65). Images were captured hourly for a period of 24 h with green fluorescent protein intensity measured using the integrated IncuCyte ZOOM image processing software. Data are shown as the average cell (green object) GFP intensity per well at 8 h posttransfection (where expression was at the maximum level).

### Statistical analysis.

Replicon mutants were compared to WT ptGFP using one-way analysis of variance (ANOVA). Differences between groups were considered to be significant at *P* values of <0.05. Error bars represent standard errors of the means (SEM) of multiple independent experiments. Statistical analyses were performed with GraphPad Prism 8.00 (GraphPad Software, San Diego, CA, USA).

### Data availability.

Full genome FMDV sequences generated as a part of this study were submitted to GenBank and are available under the following accession numbers: MW355668 to MW355680.

10.1128/mSphere.00015-21.7 FIG S7 Replication kinetics of FMDV replicon constructs containing the stem-loop mutants that were described in the legend to Fig. 7. IncuCyte data represent the average cell (green object) GFP intensity per well over a period of 12 h within BHK-21 cells and MDBK cells. Results are the means of three independent experiments ± standard errors. Download FIG S7, PDF file, 0.2 MB. © Crown copyright 2021. 2021 Crown https://creativecommons.org/licenses/by/4.0/ This content is distributed under the terms of the Creative Commons Attribution 4.0 International license.
