# Supplementary material for: Mutagenesis Mapping of RNA Structures within the Foot-and-Mouth Disease Virus Genome Reveals Functional Elements Localized in the Polymerase (3Dpol)-Encoding Region
Source: mSphere. 2021 Jul 14;6(4):e00015-21. doi: 10.1128/mSphere.00015-21 (PMC8386395; doi:10.1128/mSphere.00015-21)
Supplement: TABLE S1 [file msphere.00015-21-st001.pdf]

Supplementary Table S1

FMDV sequences selected from GenBank

Serotype:	Number of isolates:	GenBank accession numbers:
A	19	AY593788, MH053305, JF749843, HM854024, HQ832580, MH053306, KM268896, AY593802, KJ608371, MH053307, AY593751, AY593754, AY593761, AY593764, AY593766, AY593767, HM854022, AY593791, AY593794
Asia 1	12	AY593795, AY687334, DQ533483, DQ989306, DQ989315, DQ989319, EF149010, EF614458, HQ632774, JF739177, KM268898, MF782478
C	6	MH053308, KM268897, MH053309, AJ133357, MH053310, AJ007347
O	21	AY593819, MH053313, MH053311, MH053312, KF112885, KJ206909, HQ632769, HQ632771, KU291242, KR401154, GU384683, KF694737, AJ539140, MH053315, JX040491, MH053317, MH053318, MH053316, KJ560291, DQ404170, KU821591
SAT 1	19	AY593838, AY593845, MH053319, AY593844, JF749860, MH053321, AY593846, AY593839, AY593842, AY593841, AY593840, MH053322, AY593843, KM268899, MH053323, MH053324, MH053325, MH053326, MH053327
SAT2	15	MH053330, MH053332, MH053328, MH053329, JX014255, MH053333, AY593849, JX014256, AY593847, MH053335, KM268900, JF749862, MH053336, MH053337, KU821592
SAT3	13	AY593853, AY593851, MH053339, MH053340, MH053344, MH053343, AY593850, KJ820999, MH053341, MH053351, KX375417, KM268901, MH053350
