# Supplementary material for: Mutagenesis Mapping of RNA Structures within the Foot-and-Mouth Disease Virus Genome Reveals Functional Elements Localized in the Polymerase (3Dpol)-Encoding Region
Source: mSphere. 2021 Jul 14;6(4):e00015-21. doi: 10.1128/mSphere.00015-21 (PMC8386395; doi:10.1128/mSphere.00015-21)
Supplement: FIG S1 [file msphere.00015-21-sf001.pdf]

Supplementary Figure S1

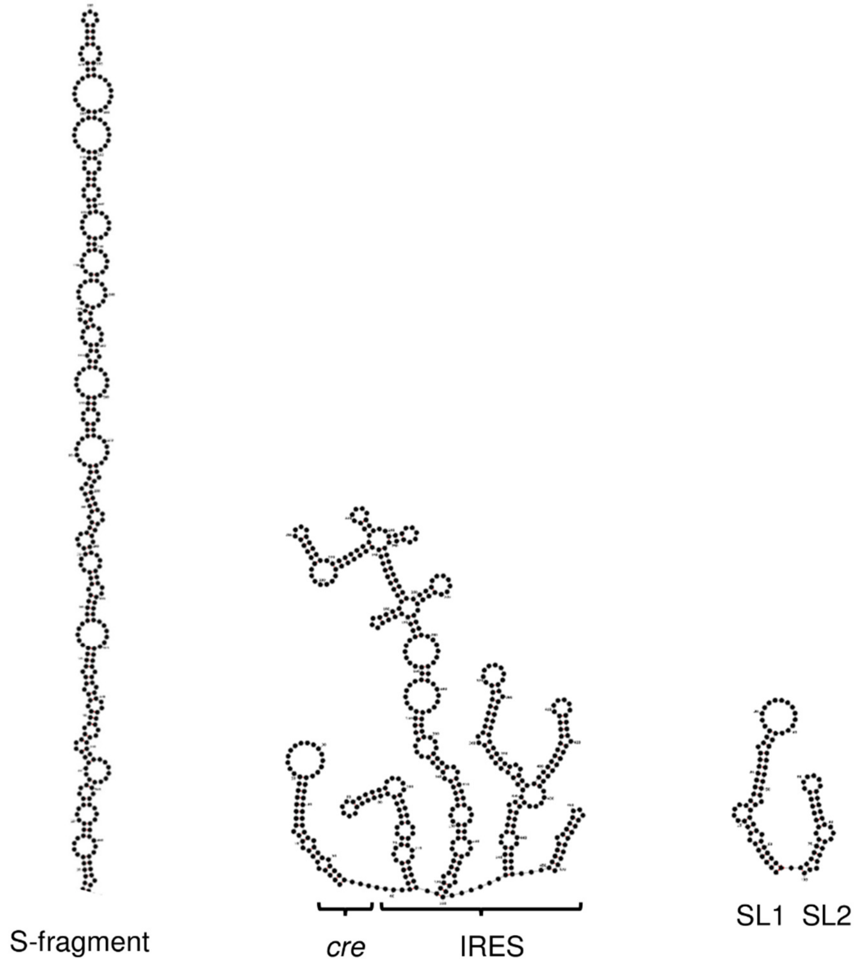

Conserved whole genome RNA structure was predicted for 118 FMDV filed isolates as described for Figure 1 and regions of previously published structures located within the 5' UTR (S-fragment, *cre* and IRES) or 3' UTR (SL1 and SL2) were visualised using Forna web service.
