# Supplementary material for: Mutagenesis Mapping of RNA Structures within the Foot-and-Mouth Disease Virus Genome Reveals Functional Elements Localized in the Polymerase (3Dpol)-Encoding Region
Source: mSphere. 2021 Jul 14;6(4):e00015-21. doi: 10.1128/mSphere.00015-21 (PMC8386395; doi:10.1128/mSphere.00015-21)
Supplement: TABLE S2 [file msphere.00015-21-st002.pdf]

Supplementary Table S2

List of the conserved stem-loops located in the ORF of FMDV genome

Stem-loop (ORF-SL)[a]	Genomic region[b]	Nucleotide position[c]	Sequence[d]	Structure[e]	Number of isolates containing ORF-SL[f]
ORF-SL1	Lab	1108-1149	UGUUUUAUCGCUUUGCUCCAGGCUUUCAGAGAGAUCAAACA	(((((.(((.((.((............)).)).))).)))))	71
ORF-SL2	Lab	1237-1271	GACAACUGUUGGCUGAACACCAUCCUGCAGCUGUU	((((.(((..((...........))..))).))))	108
ORF-SL3	Lab	1377-1399	CGAGGGUGGGCCACCCGCUCUCG	(((.(((((.....))))).)))	118
ORF-SL4	Lab-1A	1695-1722	GGGAGCCGGGCAAUCCAGCCCGGCCACC	((..(((((((......)))))))..))	100
ORF-SL5	1A	1747-1783	ACUGGUAGCAUCAUCAACAACUACUACAUGCAGCAGU	((((...((((................))))..))))	115
ORF-SL6	1B -1C	2571-2632	CGUGCACGUGGCGGGUGAGCUGCCCUCGAAAGAGGGGAUCAUCCCCGUGGCCUGUGCCGACG	(((....(((((.((.((...((((........))))....)))).))))).........)))	98
ORF-SL7	1C	2892-2907	GUACUACACACAGUAC	(((....(((((.((.((...((((........))))....)))).))))).........)))	115
ORF-SL8	2A-2B	3910-3983	UUCGACCUGCUCAAGUUGGCCGGAGACGUUGAGUCCAACCCUGGGCCCUUCUUCUUCUCCGACGUCAGGUCGAA	((((.((((.....(((((.((((((......((((((....)))))......)))))))))))..)))).))))	117
ORF-SL9	2B	4041-4069	CGGACCCGACUUCAACCGGUUGGUGUCCG	((((((((............))).)))))	118
ORF-SL10	2B	4102-4135	AUCAGGGACGGUCUCGACGAGGCCAAGCCCUGGU	((((((...((((((...))))))....))))))	117
ORF-SL11	2B	4196-4208	AGGACCCAGUCCU	(((((...)))))	118
ORF-SL12	2B	4240-4298	GAGAUUCUGGACAGCACCUUCGUCGUGAAGAAAAUCUCCGACUCGCUCUCCAGUCUUUU	((((..(((((.(((......((((.((.((...))))))))..))).)))))..))))	114
ORF-SL13	2B	4308-4356	GGCCCCUGUCUUCAGUUUCGGAGCCCCGAUUCUGUUGGCAGGGUUGGUC	((((((.(((..(((..((((....))))..)))..))).))...))))	117
ORF-SL14	2B	4365-4401	CUCGAGUUUCUUCCGGUCCACACCCGAAGACCUGGAG	(((((...(((((.(((....))).)))))..)))))	118
ORF-SL15	2B-2C	4403-4419	GAGCAGAGAAACAGCUC	((((.........))))	115
ORF-SL16	2C	4445-4466	UCGCCAUUCUCAAGAACGGCGA	(((((............)))))	118
ORF-SL17	2C	4483-4517	AUCCUGGCUAUCCGCGACUGGAUCAAAGCAUGGAU	((((.(((((((((....)))))...)))).))))	117
ORF-SL18	2C	4693-4787	GCACCAGCACCCAGCAAGUCGAGACCCGAACCCGUGGUCGUUUGCCUCCGCGGCAAAUCCGGCCAGGGCAAGAGUUUCCUUGCGAACGUGCUCGC	((...(((((...(((((..(((((.....(((.(((((((((((((.....))))))..))))))))).....))))))))))....))))).))	117
ORF-SL19	2C	4849-4894	CCUGACCACUUCGACGGUUACAACCAACAGACCGUUGUUGUGAUGG	((....(((..((((((((..........))))))))..)))..))	116
ORF-SL20	2C	4918-4941	GGCAAGGACUUCAAGUACUUCGCC	(((.((((((...))).))).)))	110
ORF-SL21	2C	4946-4957	UGGUCUCAACCA	((((....))))	102
ORF-SL22	2C	4968-5130	CCCGCCCAUGGCCUCACUCGAAGACAAGGGCAAACCUUUCAACAGCAAGGUCAUCAUUGCGACCACCAACCUGUACUCGGGGUUCACCCCGAGGACGAUGGUGUGCCCUGACGCUCUGAACCGAAGGUUUCACUUUGACAUUGACGUGAGUGCCAAGGACGGG	((((.((.((((((((((((...(((((...(((((((...(((....(((......(((((((.......((.(((.((.......))))).))..))))).))...)))...)))....)))))))...)))))...)))).))))).)))).)).))))	114
ORF-SL23	2C	5172-5202	GGACACCCACACCAACCCGGUGGCAAUGUUC	(((((((..............))...)))))	110

ORF-SL24	2C	5212-5258	UGUGCCCUUCUCAACGGCAUGGCCGUUGAAAUGAAGAGACUGCAACA	(((((.(((((((((((...))))))))...))))).....)).)))	107
ORF-SL25	2C	5274-5321	UCAACCACCUCUCCAGAACGUGUACCAACUGGUUGAGGAGGUGAUUGA	((((.((((..(((.(((((.((....)).)))))).))))))).))))	117
ORF-SL26	2C	5324-5357	GGGUGAAGCUCCACGAGAAAGUGUCGAGCCACCC	((((...(((((((......)))..)))).))))	95
ORF-SL27	3A	5392-5429	UCUGUGCUGUACUUCCUCAUUGAGAAAGGACAGCACGA	..((((((...(((.(((...))).)))...)))))).	116
ORF-SL28	3A	5597-5699	UCCGCGAGACUCGCAAGAGACAGAAGAUGGUGGAUGAUGCAGUGAAUGAGUACAUUGAGAAGGCAAACAUCACCACAGAUGACAAGACUCUUGACGAGGCGGA	(((((....(((((((((........((((((....(((....((((....))))......)))....))))))).............)))))).)))))))))	117
ORF-SL29	3A	5708-5748	CUCUGGAGACCAGCGGCGCCACCACUGUUGGCUUCAGAGAG	((((.(((((((.(((........))).))))))))..))))	118
ORF-SL30	3A	5755-5814	CUCCCAGGGCACAAGGCGGGCGAUGACGUGAACUCCGAGCCCGCCAAACCCGUGGA_GAG	((((..(((.....(((((((...((.......))...)))))))...)))..))...))	101
ORF-SL31	3C	6044-6089	GUGGUGCCCCACCGACCGACUUGCAAAAGAUGGUCAUGGCCAACAC	(((..((((....(((((.(((....))).))))))..)))))..)))	111
ORF-SL32	3C	6094-6117	CCUGUUGAGCUCAUCCUCGACGGG	(((((((((......)))))))))	118
ORF-SL33	3C	6122-6145	CAGUUGCCAUCUGCUGCGCUACUG	(((((((..........)))))))	117
ORF-SL34	3C	6149-6231	UGUUUGGGACUGCCUACCUCGUGCCUCGUCAUCUUUUCGCAGAGAAGUAUGACAAGAUCAUGUUGGACGGCAGAGCCAUGACA	((((.((..(((((..((.((((.((.(((..((((((...))))))...))).))..))))..))..)))))..))..))))	114
ORF-SL35	3C	6382-6417	CCCGUCGUUGGCGUGAUCAACAACGCCGACGUCGGG	((((.(((((((((........))))))))).))))	118
ORF-SL36	3C	6539-6557	CCGUUCUCGCAAAGGACGG	(((((((.....)))))))	110
ORF-SL37	3C	6559-6579	GCCGAGACAUUCAUCGUCGGC	(((...((.......)).)))	106
ORF-SL38	3D	6986-7016	UCAAGGGCGUCGACGGACUCGACGCCAUGGA	((...((((((((.....))))))))...))	118
ORF-SL39	3D	7021-7074	GACACCGCACCCGGUCUCCCCUGGGCCCUCCAGGGGAAACGCCGCGGAGCUCUC	((...(((.((((((.(((((((((...))))))))))...))))).)).))).))	116
ORF-SL40	3D	7076-7112	UCGACUUCGAGAACGGCACUGUCGGACCCGAGAUUGA	(((((((((....((((...))))....)))))))))	114
ORF-SL41	3D	7165-7194	UUCCUGAAGGACGAGAUUCGCCCGAUGGAG	((((....((.(((...))).))...))))	116
ORF-SL42	3D	7198-7243	GUACGUGCCGGCAAGACUCGCAUUGUCGACGUCCUGCCUGUUGAAC	((.((.((.((((.((((((......))).))).)))))).))))).))	118
ORF-SL43	3D	7245-7266	CAUCCUCUACACCAGAAUGAUG	((((.(((.....)))..))))	114
ORF-SL44	3D	7280-7293	GUGCACAAAUGCAC	((((......))))	115
ORF-SL45	3D	7299-7331	CAACGGACCGCAAAUUGGCUCGGCGGUCGGUUG	((((.((((((...........)))))).))))	117
ORF-SL46	3D	7347-7421	UUGGCAAAGAUUUGGCACACAUUUUGCCCAGUACAGAAACGUGUGGGACGUGGACUAUUCGGCCUUUGAUGCUAA	((((((..((...(((........((((((..((......)).))))).))...........))).))..))))))	98*
ORF-SL47	3D	7449-7542	GUUUGAGGAGGUGUUCCGCACGGAAUUUGGAUUCCACCCGAACGCUGAGUGGAUUCUGAAAACUCUCGUGAACACGGAACACGCCUACGAGAAC	((((....((((.(((((((.(((.((.((..(((((.((.....)).)))))..))...)).))))).......)))))...))))....))))	113
ORF-SL48	3D	7624-7647	GUGCUCUACGCCCUGCGUAGACAC	(((.(((((((...))))))))))	118
ORF-SL49	3D	7671-7705	CUACACCAUGAUUUCCUACGGGGAUGACAUCGUGG	((((...(((.(((((...)))))...))).))))	118
ORF-SL50	3D	7720-7757	GAUUUGGACUUUGAAGCUCUCAAGCCUCACUUUAAAUC	(((.((((...((..(((....)))..)).)))).)))	108
ORF-SL51	3D	7887-7914	GAUGGCUUCGAAGACCCUCGAAGCUAUC	(((((((((((......)))))))))))	118
ORF-SL52	3D	7966-7999	GGACUCGCCGUCCACUCUGGACCUGACGAGUACC	(((((((..((((.....))))....))))).))	118

ORF-SL53	3D	8000-8039	GGCGUCUCUUCGAGCC CUUCCA-GGGCCUCUUUGAGAUUC C	((.(((((......(((......)))........))))).))	117

[a]Stem-loops were called open reading frame stem-loop (ORF-SL) 1 – 53;

[b]In some cases a stem-loop may be formed by sequence of two neighbouring genomic regions (e.g., Lab-VP4); 1A, 1B, 1C and 1D, are also called VP4, VP2, VP3 and VP1, respectively;

[c]Nucleotide positions corresponding to the sequence of A/Brazil/1979 isolate (GenBank accession number AY593788);

[d]Consensus sequence obtained from alignment of 118 sequences;

[e]The conserved secondary structure is shown in dot-bracket notation;

[f]Nuber of FMDV isolates out of 118 tested;

*A shorter version of this stem-loop containing 26 nts was present in 117 of FMDV isolates.
