# Supplementary material for: Mutagenesis Mapping of RNA Structures within the Foot-and-Mouth Disease Virus Genome Reveals Functional Elements Localized in the Polymerase (3Dpol)-Encoding Region
Source: mSphere. 2021 Jul 14;6(4):e00015-21. doi: 10.1128/mSphere.00015-21 (PMC8386395; doi:10.1128/mSphere.00015-21)
Supplement: FIG S2 [file msphere.00015-21-sf002.pdf]

# Supplementary Figure S2

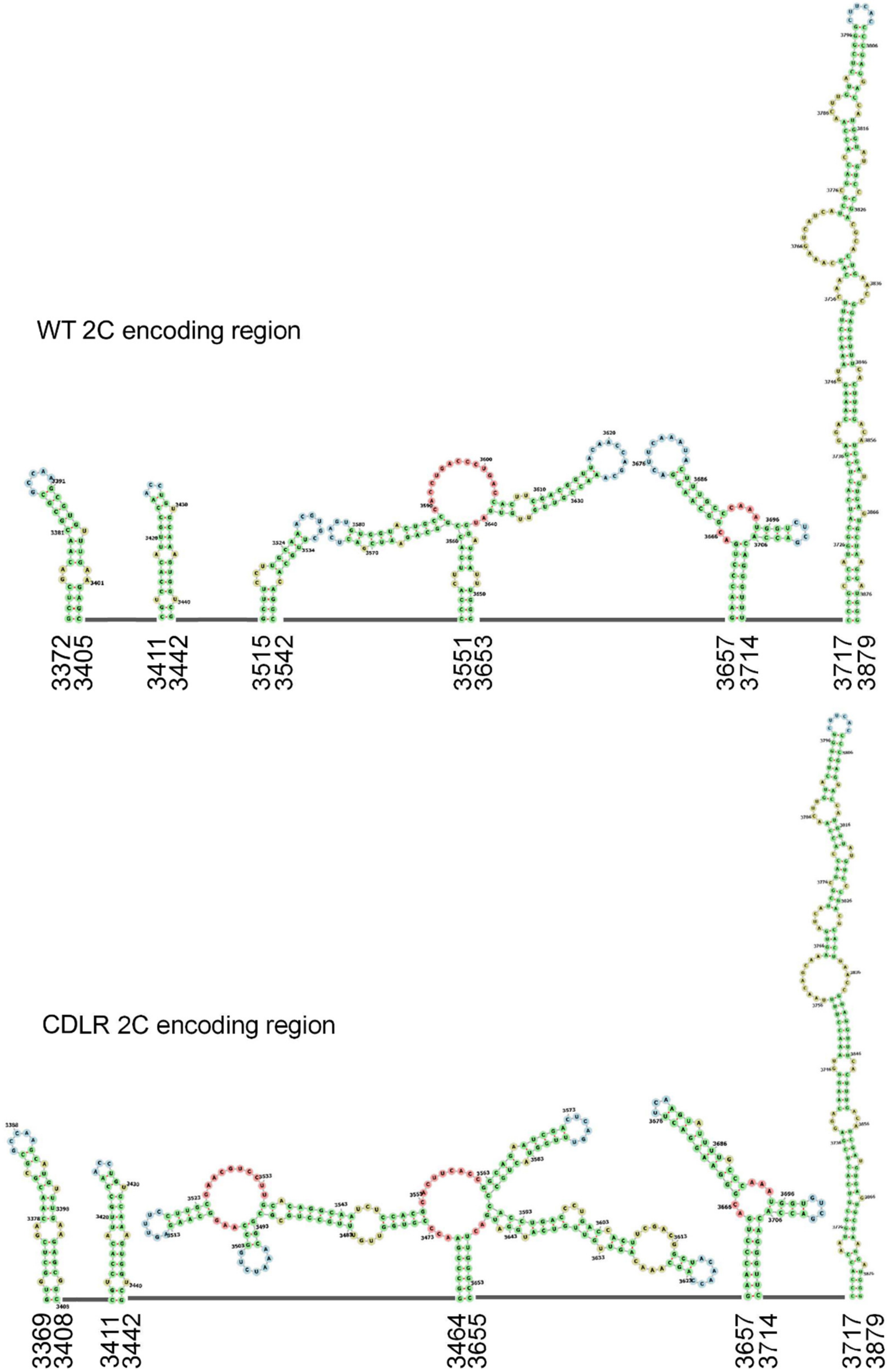

RNA structure for the whole genome of the WT replicon and a replicon with the 2C encoding region permuted by the CDLR algorithm was predicted using RNAfold. The predicted structure of the 2C encoding fragment was visualised using Forna web service. Lines represent unstructured regions and numbers at the beginning and the end of each individual stem-loop correspond to the genomic positions of each replicon. Nucleotides forming stems are shown in green, interior loops in yellow, junctions in red and hairpin loops in blue.
