# Supplementary material for: Mutagenesis Mapping of RNA Structures within the Foot-and-Mouth Disease Virus Genome Reveals Functional Elements Localized in the Polymerase (3Dpol)-Encoding Region
Source: mSphere. 2021 Jul 14;6(4):e00015-21. doi: 10.1128/mSphere.00015-21 (PMC8386395; doi:10.1128/mSphere.00015-21)
Supplement: FIG S3 [file msphere.00015-21-sf003.pdf]

Supplementary Figure S3

WT SL1 and SL2

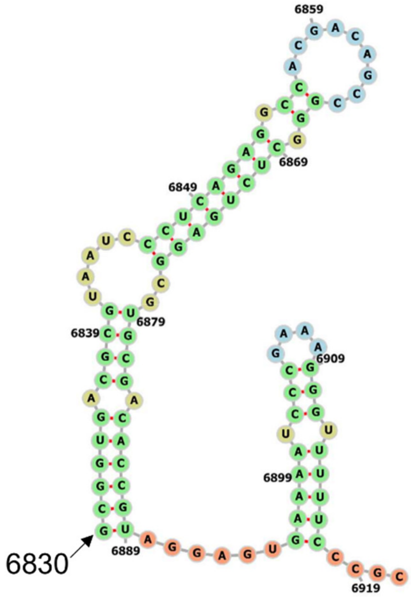

CDLR SL1 and SL2

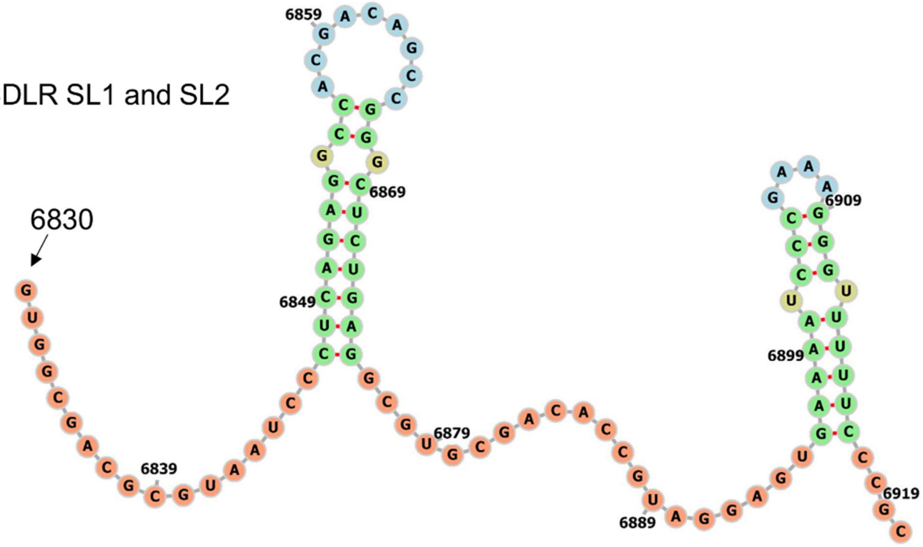

RNA structure for the whole genome of the WT replicon and a replicon with the Δ1D-3D$_3$ encoding region permuted by the CDLR algorithm was predicted using RNAfold. The predicted structure of SL1 and SL2 stem-loops located in the 3' UTR was visualised using

Forna web service. Numbers correspond to the genomic positions of each replicon. Nucleotides forming stems are shown in green, interior loops in yellow, hairpin loops in blue and unpaired region in orange.
