# Supplementary material for: Mutagenesis Mapping of RNA Structures within the Foot-and-Mouth Disease Virus Genome Reveals Functional Elements Localized in the Polymerase (3Dpol)-Encoding Region
Source: mSphere. 2021 Jul 14;6(4):e00015-21. doi: 10.1128/mSphere.00015-21 (PMC8386395; doi:10.1128/mSphere.00015-21)
Supplement: FIG S4 [file msphere.00015-21-sf004.pdf]

Supplementary Figure S4

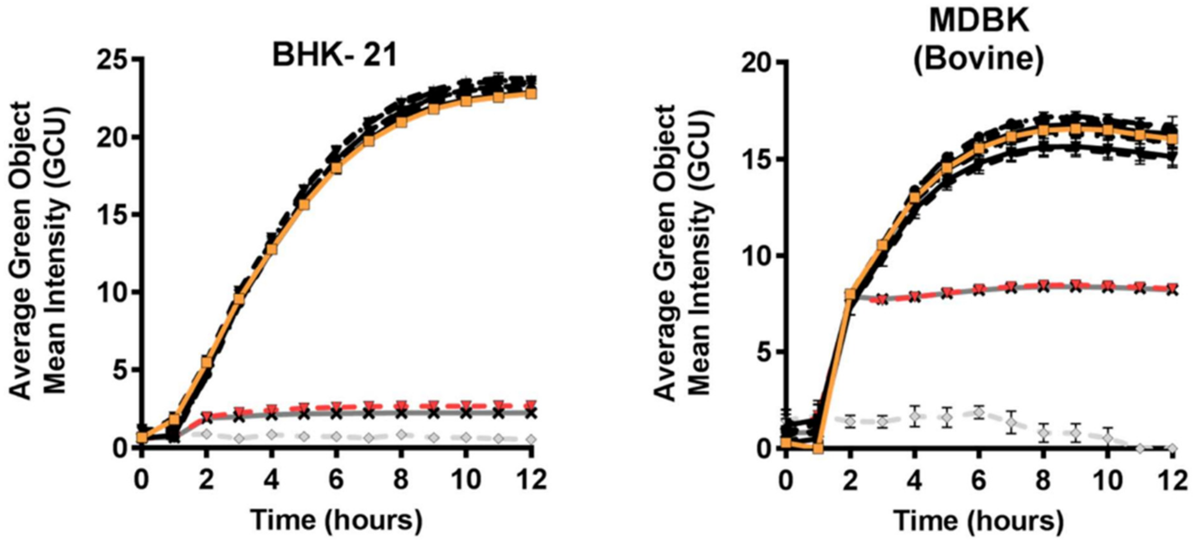

Replication kinetics of FMDV replicon constructs containing CDLR-permuted regions which were described in Figure 3. IncuCyte data represent the average cell (green object) GFP intensity per well over a period of 12 h. Replicon RNA from CDLR mutants (black (or red for $3D_3$)), WT ptGFP (orange) or ptGFP-GNN (grey) was introduced into BHK-21 or MDBK cell monolayers. Results are the mean of three independent experiments ± standard error.
