# Supplementary material for: Mutagenesis Mapping of RNA Structures within the Foot-and-Mouth Disease Virus Genome Reveals Functional Elements Localized in the Polymerase (3Dpol)-Encoding Region
Source: mSphere. 2021 Jul 14;6(4):e00015-21. doi: 10.1128/mSphere.00015-21 (PMC8386395; doi:10.1128/mSphere.00015-21)
Supplement: FIG S5 [file msphere.00015-21-sf005.pdf]

Supplementary Figure S5

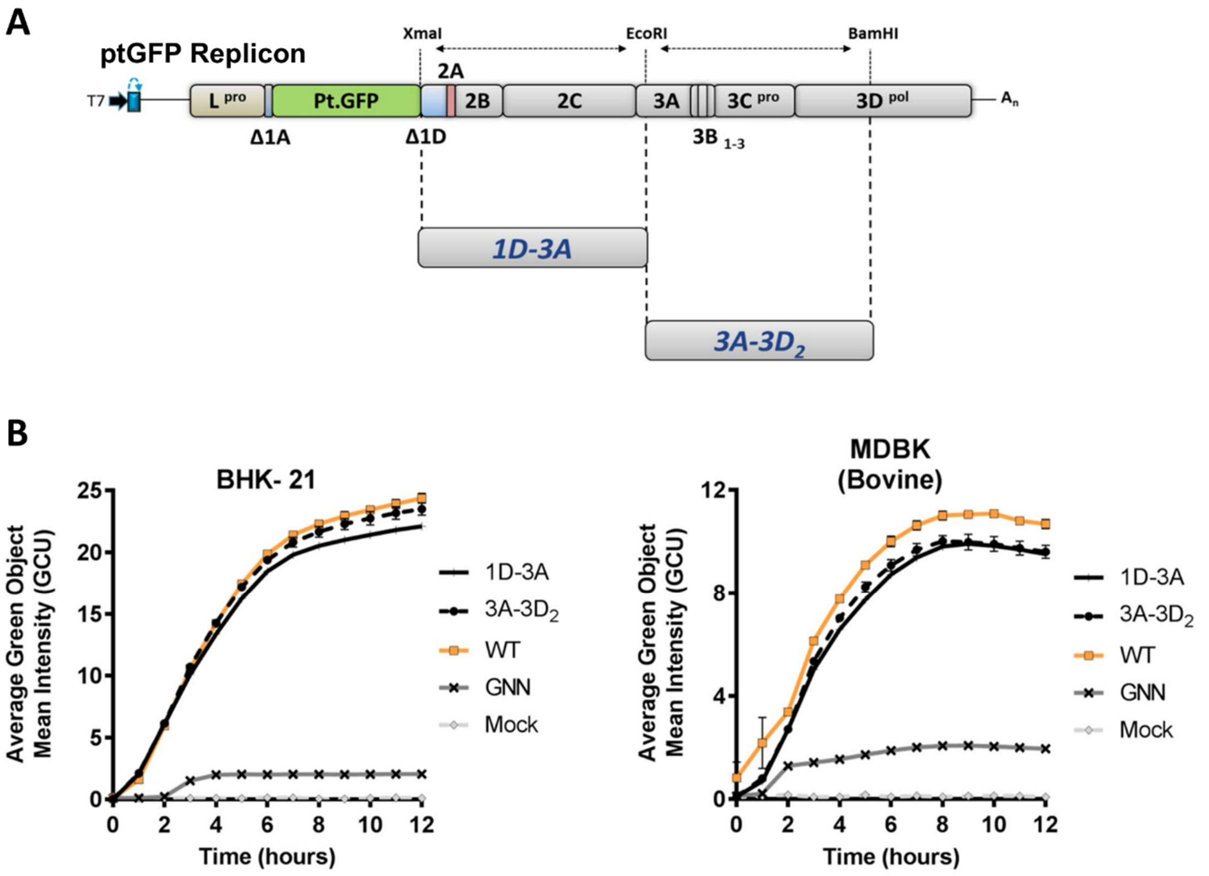

Replication kinetics of FMDV replicons containing larger sections of the genome permuted by the CDLR algorithm. **(A)** Schematic representation of CDLR replicons encoding larger mutated regions. Mutated inserts were cloned directly into the ptGFP replicon using the unique restriction enzymes shown. **(B)** IncuCyte data represent the average cell (green object) GFP intensity per well over a period of 12 h within BHK-21 and MDBK cells. Results are the mean of three independent experiments ± standard error.
