# Supplementary material for: Mutagenesis Mapping of RNA Structures within the Foot-and-Mouth Disease Virus Genome Reveals Functional Elements Localized in the Polymerase (3Dpol)-Encoding Region
Source: mSphere. 2021 Jul 14;6(4):e00015-21. doi: 10.1128/mSphere.00015-21 (PMC8386395; doi:10.1128/mSphere.00015-21)
Supplement: TABLE S3 [file msphere.00015-21-st003.pdf]

Supplementary Table S3

Supplementary legend for Figure 4A

	Pairings		
	Compatible[a]		Incompatible
**Colour**	**Number of pairings**	**Number of pairing types**	**Number of pairings**
Dark red	118	1	0
Red	117	1	1
Pink	116	1	2
Orange	118	2	0
Yellow	117	2	1
Pale yellow	116	2	2
Dark green	118	3	0
Green	117	3	1
Pale green	116	3	2
Dark turquoise	118	4	0
Turquoise	117	4	1
Pale turquoise	116	4	2
Dark blue	118	5	0
Blue	117	5	1
Pale blue	116	5	2
Grey	≤115	≤6	≥3

[a]Compatible pairings are G-C, C-G, A-U, U-A, G-U and U-G;

White = Unpaired nucleotides within loops;

○ = indicates nucleotide positions where a substitution resulted in an alternative compatible pair;

Grey bars indicate unstructured regions between the stem-loops (not drawn to scale).
